# Pharmacological targeting of BMAL1 modulates circadian and immune pathways

**DOI:** 10.1038/s41589-025-01863-x

**Published:** 2025-03-25

**Authors:** Hua Pu, Laura C. Bailey, Ludwig G. Bauer, Maria Voronkov, Matthew Baxter, Kilian V. M. Huber, Sepideh Khorasanizadeh, David Ray, Fraydoon Rastinejad

**Affiliations:** 1 Nuffield Department of Medicine, Target Discovery Institute, University of Oxford, Oxford, UK; 2 Radcliffe Department of Medicine, Oxford Centre for Diabetes, Endocrinology and Metabolism, University of Oxford, Oxford, UK; 3 NIHR Oxford Biomedical Research Centre, John Radcliffe Hospital, Oxford, UK; 4 Nuffield Department of Medicine, Centre for Medicines Discovery, University of Oxford, Oxford, UK

**Keywords:** Circadian rhythms, Biophysical chemistry, Small molecules, Target identification, Pharmacology

## Abstract

The basic helix–loop–helix PER-ARNT-SIM (bHLH-PAS) proteins BMAL1 and CLOCK heterodimerize to form the master transcription factor governing rhythmic gene expression. Owing to connections between circadian regulation and numerous physiological pathways, targeting the BMAL1–CLOCK complex pharmacologically is an attractive entry point for intervening in circadian-related processes. In this study, we developed a small molecule, Core Circadian Modulator (CCM), that targets the cavity in the PASB domain of BMAL1, causing it to expand, leading to conformational changes in the PASB domain and altering the functions of BMAL1 as a transcription factor. Biochemical, structural and cellular investigations validate the high level of selectivity of CCM in engaging BMAL1, enabling direct access to BMAL1–CLOCK cellular activities. CCM induces dose-dependent alterations in PER2–Luc oscillations and orchestrates the downregulation of inflammatory and phagocytic pathways in macrophages. These findings collectively reveal that the BMAL1 protein architecture is inherently configured to enable the binding of chemical ligands for functional modulation.

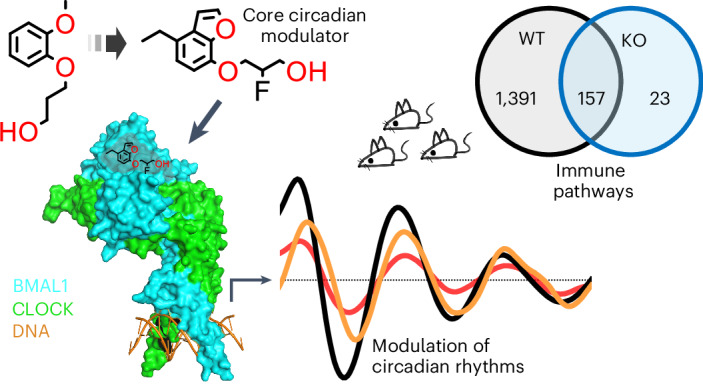

## Abstract (web summary)

The authors developed a small molecule targeting the circadian regulator BMAL1, enabling dose-dependent modulation of gene expression for circadian-controlled genes and downregulation of inflammatory pathways in macrophages.

## Main

Circadian rhythms, oscillations with an approximately 24-h period, govern a wide array of physiological and behavioral functions. At the cellular level, these rhythms are controlled by transcription–translation feedback loops (TTFLs)^1–5^. The central element of this circuitry involves brain and muscle arnt-like 1 (BMAL1, also known as ARNTL or MOP3) and circadian locomotor output cycles kaput (CLOCK), which heterodimerize to form a transcription factor^6–9^. BMAL1–CLOCK drives expression of two feedback loops. The first comprises *Periods* (*PER1–3*) and *Cryptochromes* (*CRY1/2*), which form complexes to repress the transcriptional activity of BMAL1–CLOCK. In addition, BMAL1–CLOCK transactivates nuclear receptor subfamily 1 group D (*NR1D1/2*, also known as *REV-ERBs*), and retinoic acid receptor–related orphan receptors (*RORs*), which compete to repress or activate *BMAL1* transcription, respectively^10–12^. The circadian clock is entrained to light cycles through a central pacemaker located in the suprachiasmatic nucleus, which is innervated from the retina, but the circadian TTFL clock in mammals is largely cell autonomous^13^.

Circadian dysregulation has been implicated in a spectrum of diseases, spanning cardiometabolic disorders, psychiatric conditions, autoimmune diseases, cancers and neurodegenerative disorders^14–21^. Pharmacological interventions targeting circadian rhythms hold promise for addressing these disorders. Although small molecules targeting CLOCK, CRYs, RORs and REV-ERBs have been reported^22–25^, the direct targeting of BMAL1 via a defined protein pocket has remained unexplored. Given its more central role in circadian regulation, the pharmacological targeting of the BMAL1–CLOCK complex could have broad-reaching effects beyond the core clock machinery. This approach offers an entry point to intervene in the fundamental transcription control underlying circadian processes while also facilitating deeper insights into the connections between circadian regulation and physiological pathways.

Within the TTFL circuitry, BMAL1 emerges as the only non-redundant component^6,26^. Although NPAS2 can partially compensate for CLOCK function in peripheral tissue clocks, BMAL1, particularly in mice, appears indispensable, with *BMAL2* expression reliant on BMAL1 (ref. ^27^). BMAL1, CLOCK and their paralogs all belong to the mammalian basic helix–loop–helix PER-ARNT-SIM (bHLH-PAS) family of transcription factors, where crystal structures of their bHLH–PASA–PASB segments have been described in some cases^2,28–32^. In the case of CLOCK, a previous study based on a docking approach identified the small molecule CLK8 binding to its bHLH segment^22^.

Our previous work explored the internal pockets located in the PASA and PASB domains of mammalian bHLH-PAS transcription factors, including BMAL1–CLOCK, finding that those domains could accommodate small molecules^29^. Such pockets may have evolved to accommodate endogenous ligands, as shown for aryl-hydrocarbon receptor (AhR) and hypoxia-inducible factor-3alpha (HIF3α)^31,33,34^. Synthetic compounds can bidirectionally modulate the transcriptional activities of AhR and HIF2α through their PAS domain pockets^35–37^. In the present study, we focused on the BMAL1 PASB domain (referred to as BMAL1(PASB)) to identify a ligand and evaluate whether the pocket could be leveraged to modulate BMAL1–CLOCK transcriptional activities.

## Results

### Compound discovery, engagement and selectivity studies

**Fig. 1:**
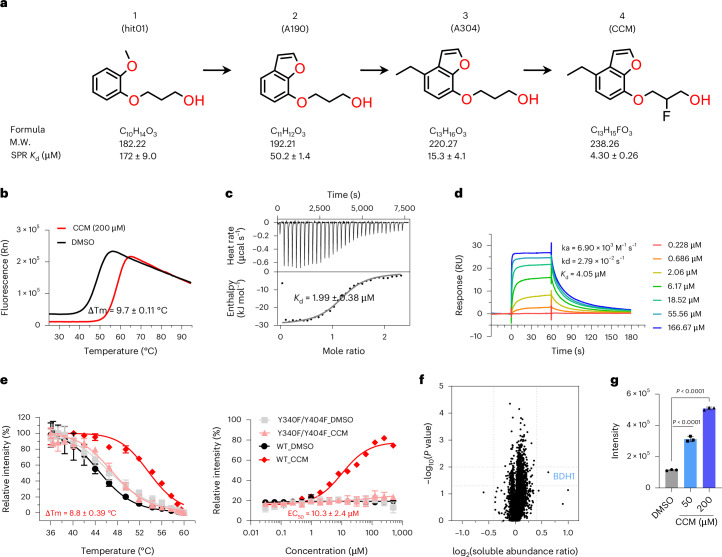
Characterization of CCM binding to human BMAL1(PASB). **a**, Hit-to-lead optimization of BMAL1 ligand. The *K*_d_ values for SPR were determined using equilibrium analysis. **b**, CCM enhances the Tm of hBMAL1(PASB) by 9.7 °C in a PTS assay. **c**, ITC detects CCM binding to hBMAL1(PASB) protein with *K*_d_ of 1.99 ± 0.38 μM, stoichiometry (*n*) value of 1.201 ± 0.028 and enthalpy (ΔH) of −29.67 ± 0.826 (kJ mol^−1^). **d**, SPR demonstrates the binding of CCM to hBMAL1(PASB) with affinity of 4.05 μM calculated from kinetic analysis. **e**, CCM stabilizes WT hBMAL1(PASB) but not Y340F/Y404F double mutant in CETSA (left). Isothermal dose–response curves of the effects of CCM on both WT and double mutant at 48 °C to obtain EC_50_ values reveal key residues necessary for compound stabilization (right). Four technical replicates were applied. Data are presented as mean values ± s.d. **f**, Volcano plot displays the thermal profile of the U2OS proteome with CCM treatment (50 μM) compared to DMSO treatment. U2OS cell lysate (*n* = 3) was heated at a temperature gradient (37–60.9 °C) for 3 min, and then denatured proteins were removed by centrifugation. The combined stabilization of the proteins in the supernatant across the heat gradient was detected by MS. The *P* value was generated by two-tailed *t*-test. **g**, CCM stabilization of HiBiT–hBMAL1(PASB) in the same U2OS cells as in **f**. The *y* axis shows its remaining protein levels after heating/denaturation steps, detected via the intensity of the Nano-Glo HiBiT signal. Error bars denote the mean ± s.d., and unpaired two-tailed *t*-test was used for statistical analyses. M.W., molecular weight; Rn, normalized reporter value. Source data

**Extended Data Fig. 1:**
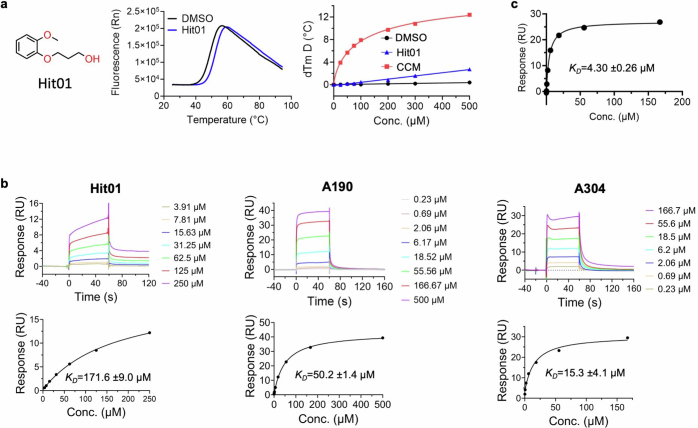
Developing small molecules for BMAL1. **a**, Structure of hit01 (Left). Melting curve of BMAL1(PASB) with DMSO or hit01 (Middle). Shift of BMAL1(PASB) Tm value with DMSO, hit01 or CCM at various concentration (Right). **b**, Affinity of compounds to BMAL1(PASB) measured by surface plasmon resonance (SPR). **c**, Affinity fitting of SPR data at equilibrium points for binding of CCM to BMAL1(PASB) protein. Source data

Using recombinant human BMAL1(PASB) protein and a high-throughput protein thermal shift (PTS) assay, which measures changes in protein stability in response to ligand binding^38^, we identified an initial hit (designated hit01, **1**) within our fragment libraries that showed promising evidence of direct binding. The screening results are contained in Supplementary Data 1. Subsequently, through hit expansion and hit-to-lead optimization, we obtained analog 190 (A190, **2**) and analog 304 (A304, **3**). Based on the structure–activity relationship, we then developed a more potent lead compound, Core Circadian Modulator (CCM, **4**) (Fig. 1a and Extended Data Fig. 1a,b). The nuclear magnetic resonance (NMR) peak listings of compounds A190, A304 and CCM are provided in the Methods section; the quality control information (NMR and mass spectrometry (MS)) and purity assessments (by high-performance liquid chromatography (HPLC)) are in Supplementary Figs. 1–3; and syntheses are described in Supplementary Fig. 4. CCM binding significantly stabilizes BMAL1(PASB) protein, as evidenced by an increased melting temperature (Tm value) of 9.7 °C, demonstrating saturable dose-dependent binding (Fig. 1b and Extended Data Fig. 1a).

We confirmed the direct binding of CCM to BMAL1(PASB) through multiple methods, including isothermal titration calorimetry (ITC). Figure 1c shows that the binding process is enthalpically driven, with an ΔH of −29.67 ± 0.826 kJ mol^−1^ and a ΔG of −31.99 kJ mol^−1^. Entropy (ΔS = 7.924 J (mol∙K)^−1^) contributes only 7.3% to the Gibbs free energy. The equilibrium binding constant (*K*_d_) measured was 1.99 ± 0.38 μM. Additionally, surface plasmon resonance (SPR) studies shown in Fig. 1d and Extended Data Fig. 1c corroborated this finding, revealing a similar *K*_d_ of 4 µM. The results from PTS, ITC and SPR established the thermodynamic basis for robust binding of CCM to the BMAL1(PASB) protein.

To determine the engagement of CCM with BMAL1 within cells, we used a BMAL1(PASB) construct with a HiBiT tag on its N-terminus and expressed it in HEK293T cells. This construct enabled the use of a cellular thermal shift assay (CETSA) to examine the dose-dependent binding and thermal stabilization of BMAL1 by CCM in the context of cellular proteins, employing split Nano Luciferase methodology^39^. Our findings revealed that CCM increased the thermal denaturation temperature of BMAL1(PASB) by 9 °C at 200 µM (Fig. 1e, left). This thermal stabilization exhibited dose dependency, with a half-maximal effective concentration (EC_50_) value of 10.3 µM (Fig. 1e, right), in line with the biochemical binding affinity (*K*_d_) range of 2–4 µM described earlier (Fig. 1c,d).

**Extended Data Fig. 2:**
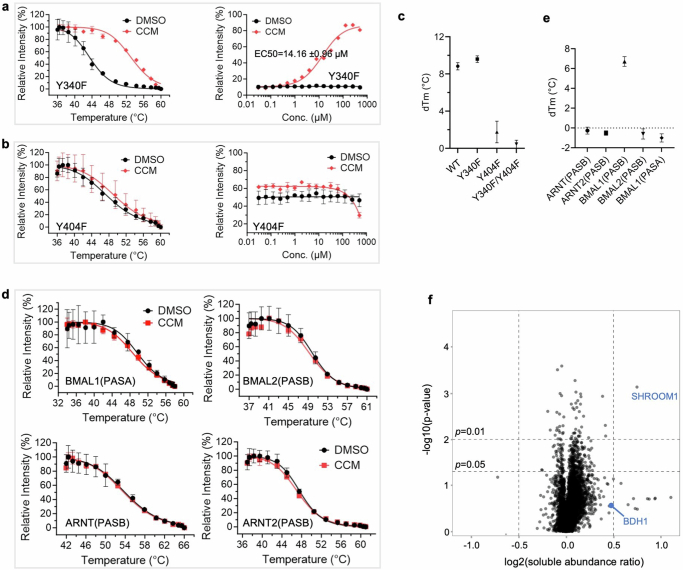
Binding specificity of CCM to BMAL1. **a** and **b**, CETSA melt curves and ITDR of the effect of CCM on mutated hBMAL1(PASB) protein Y340F (**a**) or Y404F (**b**). Four technical replicates were applied, and all error bars are presented as mean values +/− SD in **a**-**e**. **c**, Tm shift values of WT or mutated hBMAL1(PASB) in CETSA at 200 μM CCM in (**a**), (**b**) and Fig. 1e. **d**, CCM does not stabilize proteins in cell lysate that are closely related to BMAL1. CETSA melt curves are shown with DMSO or 200 μM CCM. Tm shift values are shown in (**e**). **f**, Thermal profile of U2OS proteome under 200 µM CCM treatment. SHROOM1 shows up as the only significantly stabilized protein. Source data

To validate that CCM binding was responsible for the cellular thermal stabilization of BMAL1, we used a BMAL1(PASB) construct with mutations replacing Tyr340 and Tyr404 with phenylalanine. These tyrosines were chosen based on their positions within our crystallographic complex with CCM, as discussed in the subsequent section. When using the double mutant, we observed no CCM-induced thermal stabilization in the mammalian cell CETSA experiment with HiBiT–BMAL1(PASB) (Fig. 1e and Extended Data Fig. 2a–c). Therefore, these internal cavity tyrosines play a crucial role in mediating the high degree of specificity for CCM binding to BMAL1(PASB).

After establishing that CCM directly engages BMAL1(PASB) in the context of whole U2OS cell proteome, we sought to evaluate the binding selectivity of CCM for BMAL1 compared to BMAL2 and other closely related PASB domains, including those of aryl hydrocarbon receptor nuclear translocator (ARNT), ARNT2 and the BMAL1 (PASA). We conducted additional CETSA studies and found no detectable binding to these PAS domains (Extended Data Fig. 2d,e).

To evaluate the proteome-wide target landscape of CCM, we conducted thermal profiling using quantitative MS in U2OS cell lysate^40,41^. Employing CCM concentrations of 50 µM and 200 µM, which are well above the *K*_d_ required for BMAL1(PASB) target engagement, allowed us to identify potential off-targets, including those with weak binding to CCM (Fig. 1f and Extended Data Fig. 2f). Encouragingly, very few proteins exhibited notable thermal stabilization in response to CCM, none of which is known to be involved in circadian control. As a positive control, HiBiT–BMAL1(PASB) itself showed clear evidence of CCM binding in the same cells (Fig. 1g). These data suggest that CCM displays a high degree of selectivity for BMAL1(PASB). Endogenous BMAL1 protein was not detected in these cells because of its low abundance. We also cannot exclude other potential off-targets, if their low abundance prevented their detection in this experiment.

### Ligand binding site and induced conformational shifts

**Fig. 2:**
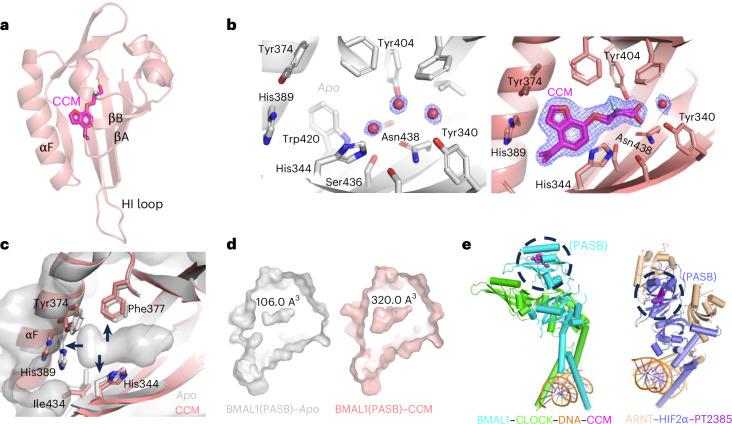
Structural basis for CCM binding to human Bmal1(PASB) and induced conformational changes. **a**, Crystal structure of BMAL1(PASB) in complex with CCM. **b**, Electron density maps in the central cavity of BMAL1(PASB), showing three water molecules in the apo structure (left) and the CCM molecule bound in the complex (right). The *R* versus *S* isomers of CCM are shown together. **c**, CCM binding repositions residues in BMAL1(PASB). **d**, CCM expands the central pocket volume three-fold. The accessible cavity size (when CCM is removed in the complex and waters are removed in the apo structure) was calculated in PyMol with PvVOL. **e**, Location of PASB domains of BMAL1 and HIF2α in their respective complexes. Structures used here are from PDB IDs 4F3L, 4H10, 4ZPK and 6E3S. The position of CCM in the complex was added manually based on the BMAL1(PAS) complex structure determined in this study.

**Extended Data Fig. 3:**
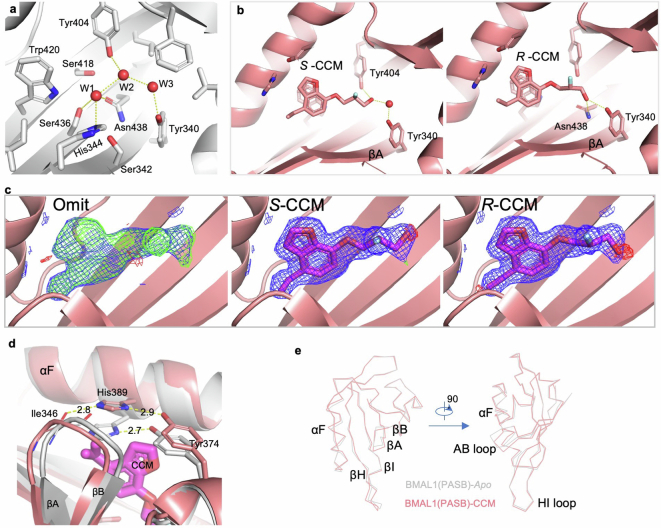
CCM-induced BMAL1 conformation changes. **a**, Three water molecules bind in hBMAL1(PASB)-*apo* structure through hydrogen-bond interaction with surrounding residues. **b**, Different binding mode for *S*-CCM and *R*-CCM in hBMAL1(PASB). **c**, Electron density maps. Left is the omit map. Center are maps with only *S*-CCM included in the model. Right are maps with only *R*-CCM included in the model. In each case, the 2*F*_o_-*F*_c_ (blue) were set at 1.0 rmsd, and *F*_o_-*F*_c_ maps (red for negative and green for positive) at 3.0 rmsd. **d**, CCM relocates strands βA and βB, in addition to helix αF. A hydrogen bond forms between His389 and the main chain of Ile346 after CCM bound. **e**, Conformation changes in the BMAL1(PASB) main chain upon CCM binding.

To visualize how CCM binds to human BMAL1(PASB), we used crystals of this protein for X-ray diffraction studies, resulting in refined structures at 1.83-Å resolution for the ligand-free (apo) state and 2.16-Å resolution for the CCM-bound BMAL1(PASB) complex. Details of the crystallographic data are in Extended Data Table 1. CCM occupies the central pocket in the BMAL1 PASB domain (Fig. 2a). In the apo structure, the central cavity houses three water molecules that interact with each other and residues Tyr340, His344, Tyr404, Ser436 and Asn438 (Fig. 2b and Extended Data Fig. 3a). Upon CCM binding, these water molecules are displaced, creating room for the compound’s 2-fluoropropanol tail moiety (‘tail’) (Fig. 2b). The tail’s primary hydroxyl group forms hydrogen bonds with residues engaged in stabilizing the water molecules in the apo structure. Trp420 and the surrounding residues create a favorable space to accommodate the benzofuran moiety of CCM. Replacing the benzene ring of hit01 with a benzofuran in CCM enables the additional π–π interactions with Tyr374 and His389, accounting for the latter’s improved binding affinity. The ethyl group and the fluorine atom of CCM, absent in A190 and A304, respectively, each occupy a small cavity in Bmal1(PASB), thereby enhancing binding affinity.

CCM features a chiral center at the C2 position of its tail. While the rigid benzofuran moiety of CCM remains stationary, the tail exhibits both *S* and *R* configurations within the pocket, binding in slightly different modes (Fig. 2b and Extended Data Fig. 3b,c). Comparison of the apo and CCM-bound structures reveals overall and locally induced changes. When looking at the details of protein, repositioning of several side chains and shifts in portions of the main chain can be found (Fig. 2c and Extended Data Fig. 3d,e). These movements include displacing His389 and shifting the position of the αF helix, where His389 is located. Induced repositioning of those and other amino acids by CCM expands the volume of the resident PASB pocket three-fold (Fig. 2d).

**Extended Data Fig. 4:**
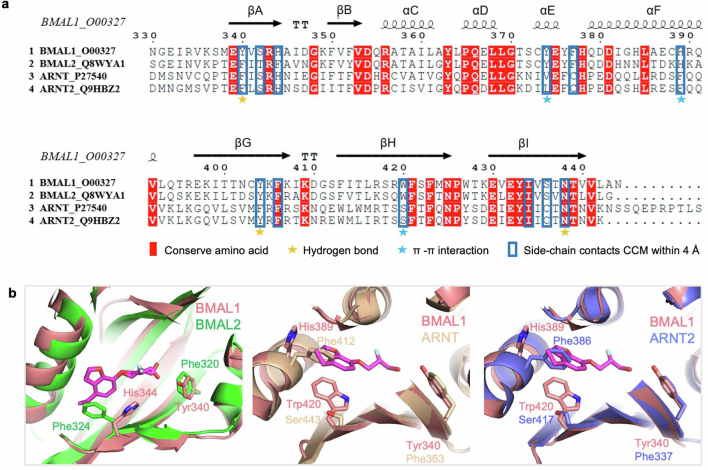
Selectivity of CCM for BMAL1. **a**, Sequence alignments of ARNT, ARNT2, BMAL1 and BMAL2. The sequence identity of PASBs to BMAL1(PASB) 338–441 are 38.5% (ARNT), 38.5% (ARNT2) and 70.2% (BMAL2). **b**, Structural basis for lack of CCM binding to related PASBs. Protein structures used here are extracted from PDB 2KDK (BMAL2), 4ZP4 (ARNT) and 7XI3 (ARNT2).

Our structural analysis of the interactions of CCM within the pocket explains its selective binding affinity for BMAL1(PASB) over highly related PASB domains. For instance, the lack of conservation in residues Tyr340 and His344 in BMAL2(PASB) explains the lack of binding (Extended Data Fig. 4a,b). Similarly, the absence of conserved residues Trp420, Tyr374 and His389 in ARNT(PASB) and ARNT2(PASB), which recognize CCM’s benzofuran group, explains the inability to bind CCM (Extended Data Fig. 4a,b). Additionally, more substantial amino acid differences between BMAL1’s PASA and PASB account for the lack of binding to the PASA domain.

### The effects of CCM on peripheral clock components

**Fig. 3:**
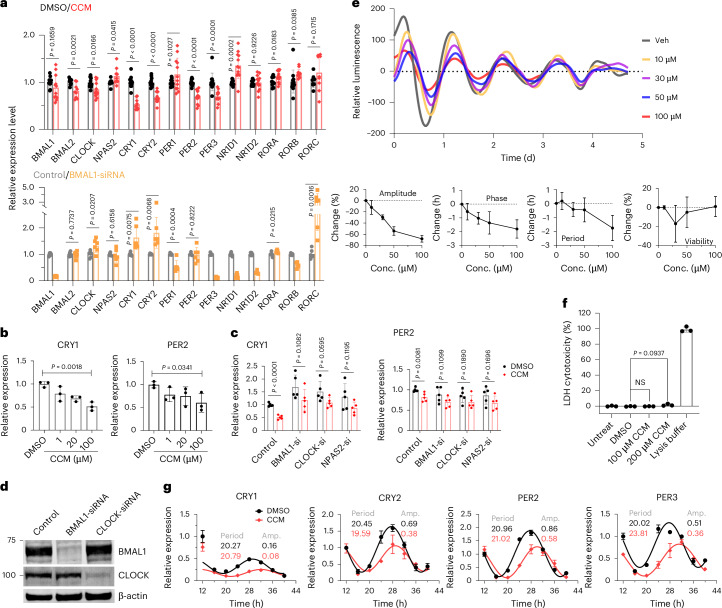
CCM modulation of BMAL1–CLOCK target gene expressions. **a**, Effect of CCM (100 μM) on target genes compared to BMAL1 knockdown. The RNA levels of BMAL1 target genes were quantified by RT–qPCR in unsynchronized U2OS cells. Six and 12 biological replicates were used for knockdown and CCM treatment, respectively. **b**, Dose-dependent effects of CCM on BMAL1 target genes in unsynchronized U2OS cells (*n* = 3, biological replicates). **c**, The effects of CCM (100 μM) are impaired when BMAL1, CLOCK or NPAS2 is knocked down in unsynchronized U2OS cells (*n* = 5, biological replicates). **d**, Validation of BMAL1 and CLOCK siRNAs by confirming reduction in their protein levels in U2OS cells. This experiment was performed independently two times with similar results. **e**, CCM dose-dependent modulation of real-time circadian rhythm observed in peritoneal macrophages directly isolated from Per2–Luc (PER2::Luc) mice (synchronized, *n* = 3–6). **f**, CCM demonstrates no significant cytotoxicity on U2OS cells (*n* = 3, biological replicates) at concentrations of 100 µM and 200 µM for 48 h. The lysis buffer provided in the CyQUANT LDH Cytotoxicity Assay Kit was used as a positive control. **g**, Monitoring of the circadian rhythm in synchronized U2OS cells (*n* = 3, biological replicates) using RT–qPCR, showing differences between DMSO vehicle (black) and 100 μM CCM treatment (red). All error bars (**a**–**c** and **e**–**g**) denote the mean ± s.d. Statistical tests were performed using unpaired two-tailed *t*-test. Amp., amplitude; Conc., concentration; NS, not significant; Veh, vehicle; RLU, relative luminescence. Source data

Our biochemical and cellular findings position CCM as a highly specific pharmacological tool for understanding the consequences of targeting this pocket in BMAL1–CLOCK functions. To assess the impact of CCM on known downstream targets within the TTFL circuitry, we conducted quantitative reverse transcription polymerase chain reaction (RT–qPCR) analysis in U2OS cells. All primers are listed in Supplementary Table 1. CCM treatment resulted in decreased expression levels of core TTFL components *CRY1*, *CRY2*, *PER2* and *PER3* while modestly increasing levels of *NR1D1*, *RORA* and *RORB* (Fig. 3a). However, *PER1*, *NR1D2* and *RORC* levels showed no significant changes with CCM treatment. Notably, the expression level of *BMAL1* itself remained unchanged after CCM treatment.

We observed dose-dependent reductions in the expression of certain BMAL1 target genes, exemplified by *CRY1* and *PER2* in Fig. 3b. Knocking down *BMAL1* attenuated the effects of CCM on these target genes (Fig. 3c), supporting the mechanism of action for requiring BMAL1. Furthermore, we compared the overall gene expression patterns induced by CCM with those resulting from *BMAL1* small interfering RNA (siRNA) knockdown in the same cells (Fig. 3a). The BMAL1 and CLOCK siRNA was validated by western blot (Fig. 3d). Interestingly, the impact of CCM across these genes was distinct from the effects of *BMAL1* knockdown. Examples include *CRY1* and *CRY2*, which are increased with knockdown of *BMAL1* but decrease with CCM. Examples further include *NR1D1*, which is reduced with knockdown but increased with CCM (Fig. 3a).

**Extended Data Fig. 5:**
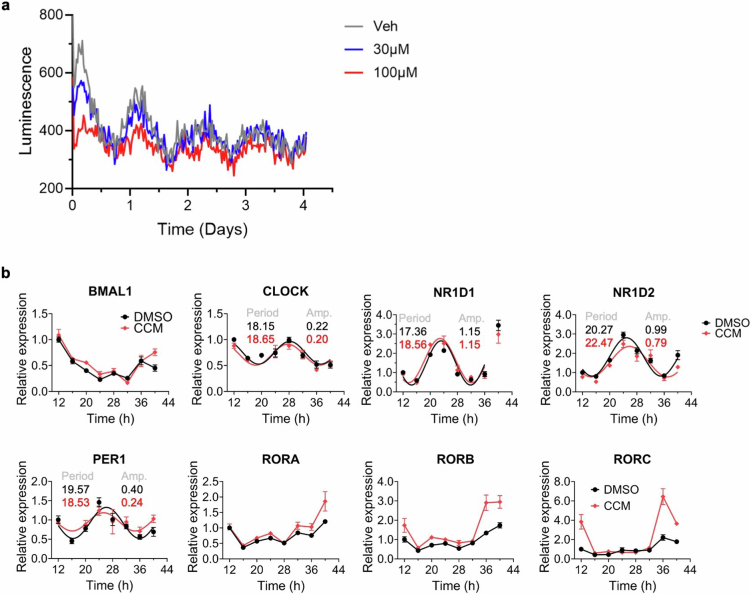
Rhythmic modulation of other BMAL1-target genes by CCM. **a**, Representative raw traces of the Per2-Luc reporter rhythms**. b**, The effects of CCM (red) versus vehicle control (black) are shown for RT-qPCR studies conducted using synchronized U2OS cells (n = 3, biological replicates). Data are presented as mean values +/− SD. Source data

Although the preceding experiments exclusively assessed CCM-induced alterations in gene expression in unsynchronized cells, we considered that CCM might also affect the amplitude and/or periods of gene expression patterns. To evaluate these potential effects on circadian gene expression patterns, we initially used synchronized peritoneal macrophages directly isolated from Per2–Luc mice. CCM significantly modified the amplitude, period and phase of the core circadian oscillator in a distinctly dose-dependent manner (Fig. 3e and Extended Data Fig. 5a). Most noteworthy were the pronounced effects on amplitude of oscillations, which were not attributable to any toxicity effects from CCM (Fig. 3e,f).

After dexamethasone-induced synchronization, we examined the CCM broader impact on circadian genes in the U2OS cell line using RT–qPCR. CCM reduced the amplitudes of *CRY1*, *CRY2*, *PER2* and *PER3* gene expression oscillations (Fig. 3g), consistent with our findings of reduced overall levels of *CRY1/2* and *PER2/3* expressions in unsynchronized cells. Interestingly, the effects of CCM remained selective for gene targets, as shown by the relatively minor changes in the cyclical expressions of other genes (Extended Data Fig. 5b).

### The effects of CCM on the macrophage gene expression signature

Numerous immune cell types, including macrophages, monocytes and neutrophils, have intrinsic circadian clock-regulated activities^42–44^. Macrophages, in particular, harbor a robust endogenous clock closely tied to their inflammatory signaling pathways^45^. Previous research demonstrated that deletion of the core clock protein BMAL1 heightened macrophage immune functions, indicating that BMAL is an immune system regulator governing phagocytic responses after infection^45^. The amino acid sequence identity of mouse and human full-length BMAL1 is 97%, and, for the BMAL1(PASB) portion, the identity is 100%. Therefore, the compound is expected to be equally effective when used with mouse macrophages as with human-derived cell lines.

**Fig. 4:**
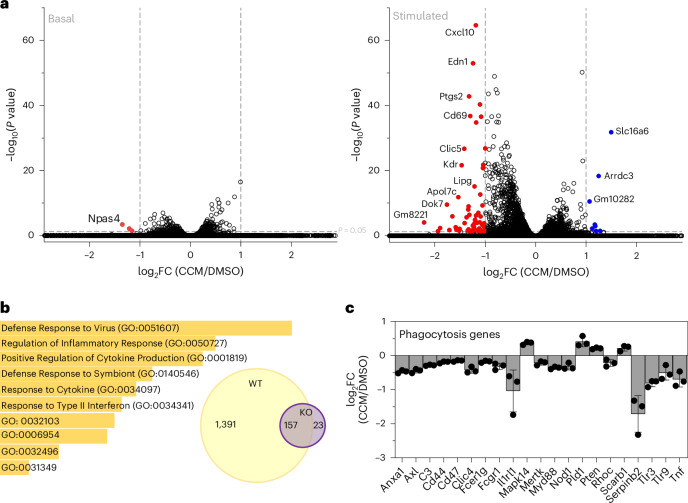
Effect of CCM treatment on macrophage gene expression signature. **a**, Volcano plots display differentially expressed genes resulting from CCM treatment in WT BMDMs in both basal and stimulated states (from treatment with LPS). Female mice were used for all conditions (*n* = 3, WT; *n* = 6, KO). Differential expression analysis used DESeq2, and *P* values were adjusted for multiple comparisons using the Benjamini–Hochberg procedure to control for FDRs. **b**, Gene Ontology (GO) for the 1,391 genes modulated by CCM in WT-stimulated macrophages. The inserted Venn diagram shows the numbers of genes regulated in the WT-stimulated macrophages, intersecting with the genes regulated in the BMAL1-KO-stimulated macrophages. **c**, Genes involved in phagocytosis are predominantly downregulated by CCM in macrophages (*n* = 3, biological replicates). Data are presented as mean values ± s.d. FC, fold change. Source data

**Extended Data Fig. 6:**
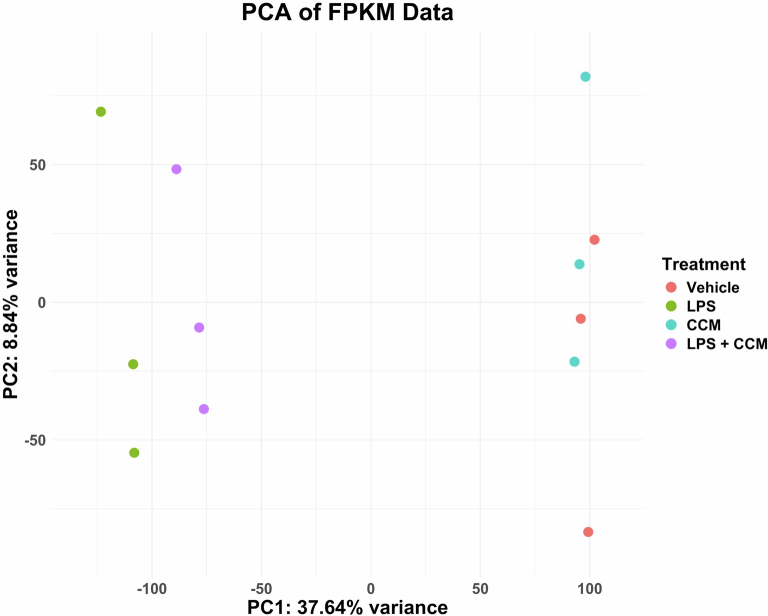
Phenotypic evaluation of macrophage after CCM treatment. Principal component analysis of the wild-type samples suggest that CCM treatment can partially revert the gene expression profile of LPS-treated cells towards the baseline profile of vehicle-treated controls. CCM alone does not significantly alter the gene expression profile compared to the control. Source data

In pursuit of a comprehensive understanding of the effects of CCM on macrophage gene expression under both basal and stimulated conditions, we employed an unbiased approach using purified bone marrow–derived macrophages (BMDMs) and RNA sequencing (RNA-seq) analysis (Fig. 4a). Under unstimulated conditions, CCM addition resulted in minimal alterations in gene expression, with only a limited number of genes showing statistical significance. Notably, among these genes was *NPAS4*, previously implicated in photonic entrainment and circadian behavior in mice^46^. However, upon stimulation of macrophages with lipopolysaccharide (LPS), the effects of CCM were significantly amplified, leading to a broader spectrum of gene expression changes. In this context, CCM inhibited a greater number of genes than it stimulated (Fig. 4a). In phenotypic evaluation, CCM alone does not have significant effect on macrophages, but CCM treatment could partially revert the gene expression profile of LPS-treated cells (M1) toward the baseline profile of vehicle-treated controls (M0) (Extended Data Fig. 6).

Although our CETSA mass spectrometric studies in U2OS cells suggested very few potential off-targets for CCM in that cell type, we seized the opportunity to further evaluate the target selectivity of CCM in macrophages. By comparing the effects of CCM on primary macrophages from wild-type (WT) mice versus BMAL1-null (*LysM-*^*Bmal1−/−*^) mice, we observed extensive regulation of multiple genes in WT macrophages, whereas a considerably smaller set of genes was affected in BMAL1-null mice, highlighting substantial target selectivity (Fig. 4b). The limited effects observed in the knockout (KO) macrophages may stem from incomplete penetrance of the gene deletion or limited off-target actions.

Upon conducting Gene Ontology analysis of the effect of CCM on BMAL1-dependent genes, we observed enrichment in functions associated with inflammation and plasma membrane processes (Fig. 4b). Given the pivotal role of phagocytosis for macrophage immune function, and its established reliance on BMAL1, our focus shifted to a selection of BMAL1 target genes associated with phagocytosis (Fig. 4c). Remarkably, CCM significantly suppressed most genes within this panel, indicating a gain of BMAL1 anti-infection function. These findings underscore the potency of CCM as a modulator of BMAL1–CLOCK activities, particularly impacting inflammatory and phagocytic pathways, and provide insights into the pharmacological implications of targeting BMAL1 within established pathways in macrophages.

We then analyzed the differences in splicing events that arise due to CCM (versus DMSO vehicle) across unstimulated and LPS conditions, transcriptome wide, to see if CCM altered transcriptional dynamics. We observed that CCM caused a dramatic increase in alternative splicing in macrophages (Extended Data Table 2). Exon skipping was identified as the predominant feature. These events were observed in WT, but not KO, cells, indicating that they are driven by BMAL1.

### CCM’s mechanism of action

**Fig. 5:**
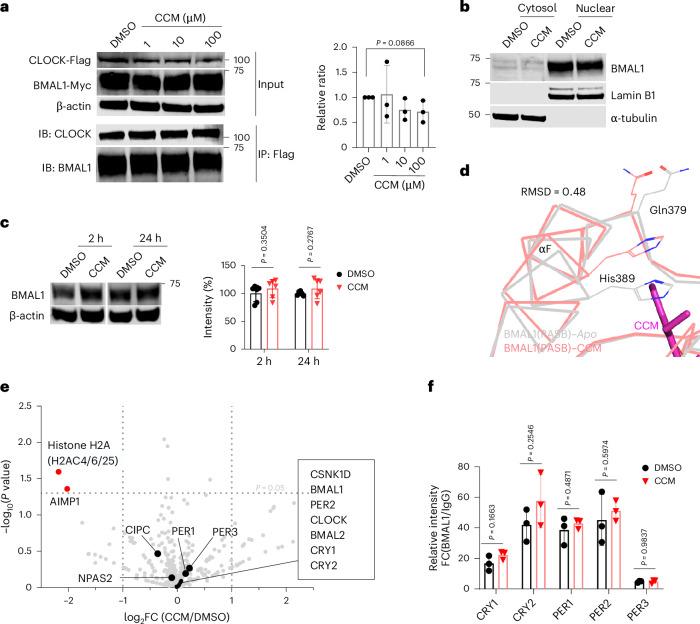
Mechanism of action studies. **a**, Co-IP studies between BMAL1 and CLOCK in U2OS cells (*n* = 3, biological replicates), demonstrating minimal effect of CCM on the stability of their complex. The ratio of BMAL1/CLOCK intensity in each IP sample was calculated and then normalized to DMSO control for each independent experiment. Three independent experiments were integrated for statistical analysis. Error bars denote the mean values ± s.d., and *P* value was generated by unpaired two-tailed *t*-test. **b**, BMAL1 localization in the nucleus of U2OS cells remains unchanged, with no observed alterations in the distribution of cytosolic versus nuclear levels after 3 h of CCM treatment at 100 μM. This experiment was performed independently two times with similar results. **c**, Overall BMAL1 protein levels in U2OS cells (*n* = 6, biological replicates), as detected by western blot, also remain unaltered after CCM treatment at 100 μM (left). BMAL1/β-actin intensity ratio was quantified (right). **d**, The PASB α-helix F and amino acids positioned on this helix, His389 and Gln379, shift positions with CCM binding. **e**, CCM weakens the association of histone H2A with BMAL1. Protein interactions of BMAL1 were analyzed by RIME. U2OS cells (*n* = 3) were treated with 100 μM CCM or 0.1% DMSO for 3 h. **f**, CCM does not change the binding of BMAL1 to the promotor of corresponding genes in U2OS cells (*n* = 3, biological replicates) detected by ChIP–qPCR. Error bars in **a**, **c** and **f** are presented as mean values ± s.d., and statistical tests were performed using unpaired two-tailed *t*-test. FC, fold change; RMSD, root mean square deviation. Source data

We began our initial investigations into the mechanisms of action for CCM by drawing from the known effects of other bHLH-PAS protein modulators. The HIF2α antagonist PT2385 uses a mechanism of action by which it allosterically impacts a crucial interaction site in HIF2α for binding to ARNT, leading to reduced heterodimer stability, inhibition of target genes and impaired ventilatory responses to hypoxia^28,35,47^. However, the positions of the PASB domains in BMAL1 and HIF2α within their respective heterodimeric complexes are varied (Fig. 2e). BMAL1’s PASB domain is situated within the crystallographically defined BMAL1–CLOCK triple domain heterodimer at the outer perimeter of the six-domain heterodimer^2^. This positioning limits the impact of CCM on overall heterodimerization stability of BMAL1–CLOCK. In contrast, the HIF2α (PASB) domain is centrally located within the HIF2α–ARNT heterodimer, facilitating PT2385-induced structural changes that reduce heterodimer stability^28,35^ In addition, our structural observations show that CCM binding does not induce alteration of BMAL1(PASB) HI-loop, responsible for interacting with the CLOCK protein (Extended Data Fig. 3e). This finding implies that the impact of CCM on the stability of the BMAL1–CLOCK heterodimer would be limited, differing from the mechanism of action of PT2385. Co-immunoprecipitation (co-IP) studies validated this expectation, confirming that CCM does not impact BMAL1–CLOCK heterodimerization in cells (Fig. 5a and Supplementary Fig. 5a,b).

We also considered several known mechanisms of action for AhR synthetic agonists and antagonists^48,49^. Small molecules interacting at the PASB domain of AhR can disrupt its binding to cytoplasmic heat shock complexes, leading to the translocation of AhR into the nucleus^33,50^. Unlike AhR, BMAL1 is not known to interact with cytoplasmic chaperone complexes. Nevertheless, we investigated whether CCM might affect the cellular distribution of BMAL1. We isolated cytosolic and nuclear fractions from U2OS cells treated with either DMSO or CCM and conducted western blot analysis. Our results showed no noticeable difference in the distribution of BMAL1 protein between compartments, with BMAL1 being predominantly located in the nuclear fraction (Fig. 5b). Furthermore, analysis of the overall cellular levels of BMAL1 protein after 2 h and 24 h of CCM treatment also revealed no significant changes (Fig. 5c).

Our structural analysis revealed that CCM-induced conformational shifts in BMAL1(PASB) include a repositioning of αF and the amino acid Gln379 (Fig. 5d). Residue Gln379 (equivalent to Gln385 in mouse BMAL1) was identified as a key contact site between BMAL1 and core histones of nucleosomes^51^. The functional significance of this residue was further validated by a mutation at this site, which altered PER2–Luc circadian oscillations, consistent with cryogenic electron microscopy (cryo-EM) studies, indicating its role in BMAL1–CLOCK-dependent chromatin opening^51^ for dynamic chromatin alterations necessary for transcriptional regulation^52^. BMAL1 was described previously as a pioneer transcription factor^52^.

To gain deeper insights into the mechanism of action, we set out to explore how CCM binding impacts BMAL1 interactions proteome wide. We took an unbiased approach, employing rapid immunoprecipitation mass spectrometry of endogenous proteins (RIME) to identify all interactions of the endogenous BMAL1 in U2OS cells (Fig. 5e). RIME is particularly suited for the study of chromatin and transcription factor complexes in a rapid and robust manner by MS^53^. Nuclei extracted from U2OS cells were lysed by sonication, and protein complexes were immunoprecipitated by anti-BMAL1 antibody or rabbit IgG. In total, 54 unique peptides were identified for BMAL1 with a sequence coverage of 71.2%. A total of 366 proteins were quantified in MS, including known BMAL1 binding proteins such as CLOCK, NPAS2, PERs and CRYs, validating the specificity of the RIME study. The interaction between BMAL1 and CLOCK proteins was not affected by CCM treatment, which confirms co-IP data in Fig. 5a.

**Extended Data Fig. 7:**
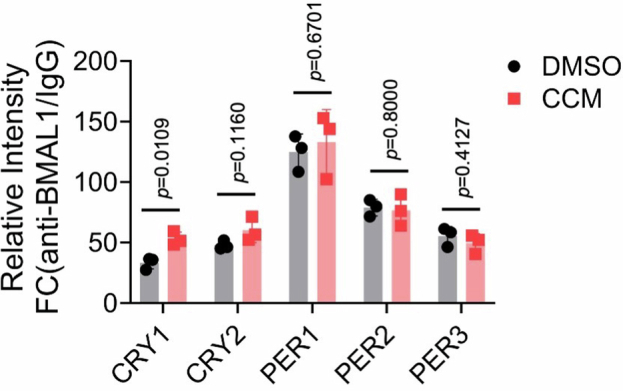
Association of BMAL1 on promotor regions of target genes. Association of BMAL1 on promotor regions of target genes as detected by ChIP qPCR in synchronized U2OS cells (n = 3, biological replicates) with CCM treatment at 100 μM for 24 h. Data are presented as mean values +/− SD. Statistical tests were performed using unpaired two-tailed *t*-test. Source data

Other known interactions with BMAL1, such as NAPS2, CRYs and PERs, also remain intact. Few proteins showed significant fold changes between CCM treatment and DMSO control (Fig. 5e). Among these, histone H2A type 1-C or 1-B/E or type 3 (H2AC6/4/25) showed the most significant change (fold change = 0.21, *P* = 0.021). We performed chromatin immunoprecipitation (ChIP) assay studies to examine the binding of BMAL1 to the promotors of CRY1/2 and PER1/2/3. As shown in Fig. 5f and Extended Data Fig. 7, CCM did not markedly alter the binding of BMAL1 protein to these sites.

## Discussion

Our studies introduce a straightforward approach to pharmacologically target the master circadian clock transcription factor comprising the central node of the TTFL, by leveraging a resident pocket in the BMAL1 PASB domain. Through complementary studies, including high-resolution structural visualization, we demonstrated that CCM exhibits direct binding to the BMAL1(PASB) pocket. The structural analysis provided a precise understanding of CCM binding mode and induced conformational changes in the protein architecture. These insights should guide future efforts in discovering and optimizing circadian modulators. The development of pharmacological tools to manipulate BMAL1 functions offers opportunities to explore its role in cellular and physiological pathways. Unlike BMAL1 knockdown, CCM treatment preserves BMAL1 protein levels while modulating its transcriptional activity as a pioneer factor. Crucially, our findings reveal that BMAL1 is inherently capable of functional modulation through the direct binding of a compound.

Our assessments of BMAL1 binding specificity consistently demonstrated a very high degree of target selectivity for CCM across various testing platforms, including proteome-wide studies. Structural comparisons of pockets among BMAL1, BMAL2, ARNT and ARNT2 revealed differences at key positions crucial for CCM binding to BMAL1, thus explaining its robust targeting. Further evaluation of CCM’s potential targets across the cellular proteome was essential for clarifying the connection between CCM’s binding to BMAL1 and the observed cellular effects reported here.

Exploring the impact of CCM on circadian rhythm regulation revealed key insights about the unique pharmacological consequences of harnessing the BMAL1 pocket. Unlike conventional agonists or antagonists previously discovered for the related bHLH-PAS transcription factors AhR and HIF2α, the effects of CCM through the PASB pocket of BMAL1 extend beyond binary classification, influencing circadian parameters in a gene-selective manner. Notably, the effects of CCM on target genes also did not align with observed effects of BMAL1 knockdown in the same cells, suggesting distinct regulatory mechanisms at play. For example, with BMAL1 knockdown, RNA levels of REV-ERBs (NR1D1 and NR1D2) decreased dramatically, whereas RORC increased. In contrast, CCM did not comparably change the levels of REV-ERBs or RORC. Similarly, for CRY1 and CRY2, BMAL1 knockdown increased their expression levels, whereas CCM treatment reduced them. The divergence between the effects of gene knockdown and pharmacological manipulation of BMAL1 can be partly attributed to feedback loops responding differently to the loss of BMAL1 protein level compared to the pharmacological modulation of its transcriptional activities, which occurs without changes in BMAL1 protein level. The compound likely affects BMAL1 chromatin function, as BMAL1 is known to be a pioneer transcription factor^52^. Together with CLOCK, it can engage nucleosomal DNA using critical interactions between the PAS domains and the core histones in the nucleosome. Changes in BMAL1 chromatin function resulting from CCM binding may manifest as alterations in histone binding, elongation dynamics and RNA alternative splicing.

Our findings also show that the binding pocket of the PASB domain exhibits remarkable adaptability, undergoing considerable expansion when binding to CCM. This expansion accompanies an induced shift in the αF helix, which is a crucial site for BMAL1–CLOCK chromatin binding. Rhythmic BMAL1–CLOCK chromatin binding and opening are known to be essential for circadian gene expression^52^. Our investigations into the effects of CCM on BMAL1 nuclear and cytoplasmic distribution, protein levels or heterodimerization with CLOCK ruled out these alternative modes of action. The RIME studies further confirmed that CCM did not alter BMAL1 interactions with CLOCK, CRYs and PERs, which are among its known cellular interaction partners.

Our RIME findings showed that CCM impacted interactions between BMAL1 and histone H2A. This result emerged from an unbiased proteomic experiment designed to identify protein candidates whose interactions with BMAL1 were altered by CCM. This finding aligns with structural observations, which demonstrated that, when CCM binds to the PASB domain of BMAL1, it induces a structural shift in the region of the protein used for chromatin binding. The outcome from the RIME study supports earlier cryo-EM studies and mutagenesis studies of Q379, which revealed that the PAS domain of BMAL1 is involved in chromatin interactions. These findings offer a coherent mechanism by which CCM influences gene expression patterns through its effects on the PASB domain’s structure and its subsequent impact on chromatin interactions. The effects of CCM were notably pronounced in reducing expression patterns and circadian amplitudes of genes strictly located inside the core circuitry, namely CRY1/2 and PER2/3.

ChIP data revealed that CCM did not substantially alter the binding of BMAL1 to its PER and CRY DNA elements. However, CCM binding led to a dramatic increase in BMAL1-dependent exon skipping events. Splicing is physically coupled to transcription and influenced by transcript elongation rates^54,55^. Exon skipping serves as an indicator of changes in overall transcriptional dynamics, suggesting that BMAL1 function may have shifted even though its DNA binding remains unchanged. Transcription factors are known to affect elongation dynamics, thereby altering splicing outcomes, such as exon skipping. There are well-established connections between chromatin regulation and RNA alternative splicing, with chromatin structure, nucleosome positioning and histones playing key roles in providing a dynamic scaffold for transcription and splicing^56,57^.

Circadian rhythms play a fundamental role in regulating numerous physiological processes, including sleep–wake cycles, hormone secretion, metabolism and immune function. Disruptions of these rhythms, whether due to genetic mutations, shift work, jet lag or lifestyle factors, are linked to numerous human diseases and health conditions. In particular, circadian amplitude has gained attention as important for healthy energy metabolism^58^ and also for intact innate immunity^59^. Aging is associated with loss of the circadian variation in macrophage phagocytic function^59^. It is likely that changes in circadian amplitude result in changes in the proportion of downstream gene targets that are captured by the circadian oscillator, resulting in altered physiology. However, the precise role of and importance of circadian amplitude remains undetermined. While our data suggest that anti-inflammatory benefits could be derived from CCM-mediated BMAL1–CLOCK pharmacological modulation, the wider set of associations between circadian disruptions and human diseases underscores the potential benefits of BMAL1–CLOCK-directed modulators.

The mechanism of action of CCM is clearly distinguished from the previously mentioned compound CLK8, which interacts with the bHLH domain of the CLOCK protein and represses the translocation of CLOCK from cytosol to nucleus^22^. Future compounds targeting BMAL1 with improved pharmacokinetics, particularly with enhanced blood–brain barrier penetration in mice, should allow for the probing of connections between pharmacological BMAL1 manipulation and alterations in whole-animal physiological responses and behaviors. Such advancements will deepen understanding of circadian regulation and drive the development of translational clock modulators. Our findings of a modulatory pocket located within the BMAL1 protein architecture also suggest that endogenous cellular ligands could impact and align the central and peripheral functions of BMAL1–CLOCK, potentially influencing circadian parameters and their synchronizations with other signaling pathways. In this context, BMAL1 and its broader bHLH-PAS transcription factor family may be likened to nuclear receptors^60–62^, with resident pockets that are configured for sensing and responding to environmental and cellular ligands.

## Methods

### Cloning, mutagenesis, expression and purification of human BMAL1(PASB)

Human BMAL1 (UniProt O00327) PASB domain (338–449 amino acids) was subcloned into pMKH vector to produce His6-TEV-hBMAL1(PASB) protein. Plasmid was transformed into Rosetta (*DE3*) cells, and a single clone was kept for further protein expression. Cells were grown in LB medium, and protein expression was induced with 0.2 mM IPTG overnight at 18 °C. Cells were harvested by centrifugation and resuspended in buffer containing 20 mM Tris-HCl pH 8.0, 500 mM NaCl, 5% glycerol, 5 mM imidazole and 1× protease inhibitor (Roche, 81410500). Cells were lysed by sonication, and protein was purified through affinity chromatography with nickel resin. The eluted fractions were then loaded on a HiLoad 16/600 Superdex 75 gel filtration column to remove aggregations and imidazole. His-tag was cleaved by overnight TEV cleavage. Tag was separated and protein was isolated by size-exclusion chromatography with a buffer of 20 mM Bis-Tris propane pH 8.0 and 150 mM NaCl, and monomer fractions were collected and concentrated to 4 mg ml^−1^ for further usage. Site-directed mutagenesis was performed with an Agilent QuikChange Kit (200522). The quality control (SDS-PAGE and MS) for BMAL1(PASB) protein is shown in Supplementary Fig. 6.

### Crystallization, structure determination and refinement

Crystals were grown in a 96-well crystallization plate (MRC96T-UVP) through sitting-drop vapor diffusion. The reservoir solution consisted of 20% PEG3350 and 0.15 M sodium formate. Protein was dissolved in 20 mM BTP pH 8.0 and 150 mM NaCl at 4 mg ml^−1^. Protein and reservoir solution was mixed in a 1:1 ratio and incubated at 4 °C for 1 month before harvesting. Compound soaking was performed 24 h before harvesting at a concentration of 1.25 mM. Diffraction datasets were collected at the Diamond Synchrotron Light Source, using beamline I03 operating at a wavelength of 0.9763 Å, with the detector Eiger2 XE 16M and the crystal temperature of 100° K. The diffraction images were indexed, merged and scaled in xia2 DIALS^63^. The initial phase was determined by molecular replacement with a model prepared from Protein Data Bank (PDB) ID 4F3L in Phaser^64^. The entire model of hBMAL1(PASB) was built manually in Coot^65^. Further structure refinements were done in CCP4 (ref. ^66^) and phenix.refine^67^. The final structures have 98.08% (BMAL1(PASB)-*Apo*) and 97.06% (BMAL1(PASB)-CCM) of residues in the favoured regions of the Ramachandran plot, with 0% outliers in both structures. Structure figures were prepared in PyMoL. Pocket sizes in the protein were examined for ligands and measured with PyVOL^68,69^. The crystal diffraction and refinement statistics are summarized in Extended Data Table 1.

### Protein thermal shift assay

Protein thermal shift assays were carried out in a 384-well plate with QuantStudio 7 Flex. Compounds were dispensed by Echo 555 liquid handler, and protein solution was added with multidrop. The protein solutions were prepared by diluting stock to 0.1 mg ml^−1^ in the buffer containing 20 mM Bis-Tris propane pH 8.0, 150 mM NaCl and 8× SYPRO Orange dye (Invitrogen, S6651). Plates were centrifuged at 1,000*g* for 30 s and incubated at room temperature for more than 10 min before measuring. Melting curves were generated by heating the plate from 25 °C to 95 °C with a speed of 0.1 °C s^−1^. Data were analyzed in Protein Thermal Shift Software version 1.4. Screening data were analyzed in DataWarrior version 6.1.0 (ref. ^70^). The terms dTm B and dTm D refer to shift of protein melting temperature (delta Tm), calculated by the Boltzmann fitting method and the derivative fitting method, respectively.

### Cell culture and viability test

Cells were grown in DMEM media (Gibco, 31966-021) supplied with 10% FBS (Gibco, A3160502) and 50 U ml^−1^ penicillin–streptomycin (P/S) (Gibco, 15070063). All cells were incubated in 5% CO_2_ at 37 °C. Medium was refreshed every 2–3 d, and transfection was done at a cell confluence of 60–80%. Transient transfections of cell lines were performed using Lipofectamine 3000 (Invitrogen, L3000-015) according to the manufacturer’s instructions. Lipofectamine RNAiMAX (Invitrogen, 13778-075) was used for siRNA knockdown experiments. siRNA oligos targeting BMAL1 (sc-38165), CLOCK (sc-35074) and NPAS2 (sc-38169) were purchased from Santa Cruz Biotechnology. Cell viability was detected with a CyQUANT LDH Cytotoxicity Assay Kit (Invitrogen, C20300). Cells were incubated in DMEM media with indicated amounts of CCM for 48 h before carrying out the viability test. Absorbances at 490 nm and 680 nm were measured in a microplate reader, and data were processed according to the manufacturer’s instructions.

### CETSA

HEK293T or U2OS cells were cultured in DMEM media, and plasmid was transfected when cells grew to 60–80% confluence. Cells were harvested 2 d after transfection. Cells were washed twice with ice-cold PBS and harvested by scraping on ice and suspended in buffer containing 20 mM Bis-Tris propane pH 8.0, 150 mM NaCl and 1× protease inhibitor cocktail (Roche, 61431800). Cells were sonicated, and supernatant of cell lysate was collected after centrifugation. Compounds were transferred into a 384-well plate through an Echo 555 liquid handler, and 5 μl of cell lysates was added to each well. Plates were centrifuged at 1,000*g* for 10 s and incubated at room temperature for 30 min. Plates were then heated in a PCR thermal cycler (Bio-Rad, C1000 Touch) for 3 min at a 36–60 °C gradient. Denatured protein was removed by centrifugation at 4 °C for 15 min at 4,300*g*. Soluble HiBiT-tag protein was detected using a Nano-Glo HiBiT Lytic Detection System (Promega, N3040) according to the manufacturerʼs protocol. Isothermal dose–response (ITDR) measurements were done at 48 °C with indicated compound concentration.

### ITC

ITC was performed in a Nano ITC (TA Instruments). Compound was dissolved directly in buffer (20 mM BTP pH 8.0 and 150 mM NaCl) and adjusted to a final concentration of 320 μM. Protein was prepared in the same buffer and diluted to 40 μM. Both protein and compound were degassed before loading onto the Nano ITC. The rotation speed was set to 180 r.p.m., and the system was kept at 20 °C. Compound was titrated into protein with an interval of 240 s. Binding data after the subtraction of the buffer titration were analyzed, and thermodynamic parameters were obtained by fitting to a one-site binding model.

### SPR

SPR was carried out in a Biacore S200 (GE Healthcare) with sensor chip SA (Cytiva, BR100531). Human BMAL1 residues 338–449 were subcloned into pET-28a vector with an N-terminal His-tag and a C-terminal avi-tag. Plasmid was then transformed into BL21 (*DE3*) competent cells that overexpressed BirA. Then, 200 nM biotin was added along with IPTG during protein expression. Final protein was purified by affinity chromatography and size-exclusion chromatography. SA chip channel was first washed with 1 M NaCl and 50 mM NaOH, and protein was then immobilized directly on the SA chip through capture with pre-immobilized streptavidin. The immobilization level was set to 4,500 response units (RU). Assay cycles were set at 20 °C using a running buffer of 50 mM BTP pH 8.0, 150 mM NaCl and 1% DMSO. A 0–2% DMSO range was used for solvent correction. The analyte was injected at 30 μl min^−1^ for 60 s with a dissociation time of 120 s. Data were fit with a one-site binding model.

### Compound synthesis

The hit01 compound in powder form was purchased from Enamine (Z425795906). Compounds A190, A304 and CCM are not commercially available. We designed these compounds using Maestro (Schrodinger Suites 2021-03), and they were synthesized by Cortex Organics Ltd. (https://www.cortexorganics.com/). The starting material for their syntheses was purchased from BLD Pharmatech (BD110688 and BD281593). Quality control data for A190, A304 and CCM, including MS and NMR spectra, are in Supplementary Figs. 1–3. The steps for their syntheses are shown in Supplementary Fig. 4.

#### The standard peak listings of CCM

1H NMR (500 MHz, CDCl_3_) δ 7.62 (d, *J* = 2.1 Hz, 1H), 6.95 (dt, *J* = 7.9, 0.8 Hz, 1H), 6.80 (d, *J* = 2.1 Hz, 1H), 6.78 (d, *J* = 8.0 Hz, 1H), 5.00 (dqd, *J* = 47.8, 5.0, 3.7 Hz, 1H), 4.43 (ddd, *J* = 20.0, 4.9, 1.9 Hz, 2H), 4.13 – 3.89 (m, 2H), 2.81 (qd, *J* = 7.5, 0.7 Hz, 2H), 1.94 (t, *J* = 6.5 Hz, 1H), 1.29 (t, *J* = 7.6 Hz, 3H); 13C NMR (126 MHz, CDCl_3_) δ 144.76, 144.42, 142.41, 130.82, 128.57, 121.66, 108.85, 105.65, 91.80 (d, *J* = 173.0 Hz), 68.38 (d, *J* = 25.4 Hz), 62.51 (d, *J* = 22.2 Hz), 25.86, 15.16; 19F NMR (58 MHz, CDCl_3_) δ −109.28 (s, 1F); LC–MS (*m*/*z*): [M + H]^+^ calculated for C_13_H_15_FO_3_, 239.1078; found, 238.978.

#### The standard peak listings of A304

1H NMR (500 MHz, CDCl_3_) δ 7.61 (d, *J* = 2.2 Hz, 1H), 6.94 (d, *J* = 8.0 Hz, 1H), 6.79 (d, *J* = 2.2 Hz, 1H), 6.76 (d, *J* = 8.1 Hz, 1H), 4.34 (t, *J* = 6.0 Hz, 2H), 3.93 (q, *J* = 5.7 Hz, 2H), 2.81 (q, *J* = 7.6 Hz, 2H), 2.13 (p, *J* = 5.9 Hz, 2H), 1.89 (t, *J* = 5.5 Hz, 1H), 1.29 (t, *J* = 7.6 Hz, 3H); 13C NMR (126 MHz, CDCl_3_) δ 144.67, 144.45, 142.96, 130.04, 128.29, 121.61, 107.99, 105.62, 67.37, 60.84, 32.23, 25.86, 15.22; LC–MS (*m*/*z*): [M + H]^+^ calculated for C_13_H_16_O_3_, 221.1172; found, 221.485.

#### The standard peak listings of A190

1H NMR (500 MHz, CDCl_3_) δ 7.62 (d, *J* = 2.1 Hz, 1H), 7.20 (dd, *J* = 7.8, 1.1 Hz, 1H), 7.14 (t, *J* = 7.8 Hz, 1H), 6.83 (dd, *J* = 7.9, 1.1 Hz, 1H), 6.76 (d, *J* = 2.1 Hz, 1H), 4.36 (t, *J* = 6.0 Hz, 2H), 3.94 (q, *J* = 5.4 Hz, 2H), 2.15 (p, *J* = 5.9 Hz, 2H), 1.95 – 1.87 (m, 1H); 13C NMR (126 MHz, CDCl_3_) δ 145.08, 144.81, 144.61, 129.40, 123.58, 113.93, 107.82, 107.04, 67.12, 60.68, 32.19; LC–MS (*m*/*z*): [M + H]^+^ calculated for C_11_H_12_O_3_, 193.0859; found, 193.071.

### Western blot and cell subfraction studies

Whole-cell lysate was directly extracted from cells with RIPA buffer or obtained by sonication. For RIPA buffer extraction, cells were harvested and suspended in RIPA buffer for 30 min with rotation and then centrifuged at 17,000*g* for 30 min to collect the supernatant (whole-cell lysate). For sonication, cell pellets were suspended in intended buffer and sonicated in an ice bath for 2 min. A final centrifuge step at 17,000*g* for 30 min was conducted to collect lysate.

To extract subcellular fraction, cells were washed twice in ice-cold PBS and then harvested by scraping. Cell pellets were centrifuged at 1,000*g* for 5 min to remove all liquid. Then, cell pellets were resuspended in hypotonic buffer (20 mM HEPES pH 7.5, 1.5 mM MgCl_2_, 10 mM KCl, 1 mM EDTA and 0.05% NP40) and incubated on ice for 15 min. The mixtures were centrifuged at 1,000*g* for 10 min, and supernatant was collected (cytosol fraction). The pellets were washed once with hypotonic buffer. All liquid was removed, and the rest was kept for further analysis (nucleus fraction).

Samples were separated in SDS-PAGE with 8% Tris-glycine gels and then transferred to PVDF membranes through the semi-dry transfer method. Membranes were first blocked in 5% BSA and then incubated overnight at 4 °C with the following primary antibodies: BMAL1 (Santa Cruz Biotechnology, sc-365645x, 1:1,000 dilution), CLOCK (Cell Signaling Technology, 5157S, 1:1,000 dilution), α-tubulin (Abcam, ab4074, 1:5,000 dilution), Lamin B1 (Santa Cruz Biotechnology, sc-374015, 1:200 dilution), β-actin (Abcam, ab184092, 1:10,000 dilution), Myc-tag (Cell Signaling Technology, 2278S, 1:1,000 dilution) and Flag-tag (Sigma-Aldrich, F4042, 1:1,000 dilution). Membranes were washed three times in PBST buffer and incubated with LI-COR secondary antibody (anti-mouse, 926-68070, 1:15,000 dilution; anti-rabbit, 926-32211, 1:15,000 dilution) for 1 h at room temperature. Membranes were washed another three times before detecting in a LI-COR Odyssey gel imager. Membranes were stripped with fluorescent western blot stripping buffer (Thermo Fisher Scientific, 62300) for reprobing.

### Co-IP

Full-length human BMAL1 or CLOCK was cloned into pCMV vector with a C-terminal Myc or Flag tag. Plasmids were transfected into U2OS cells for transient expression. After 2 d, U2OS cells in 150-mm dishes were then treated with 0.1% DMSO or 1–100 µM CCM for approximately 2.5 h. Cells were then washed twice in ice-cold PBS and harvested by scraping. Then, 1.0 ml of buffer (20 mM HEPES pH 7.5, 100 mM NaCl, 1 mM EDTA, 3% glycerol, 0.1% Tween 20, 0.05% Triton X-100, 1 mM DTT and 1× cocktail protease inhibitor) was added to suspend cell pellets. Additional DMSO/CCM was added into the buffer to maintain in-cell interactions. Lysate was obtained through sonication and centrifugation. Magnetic Flag beads (Millipore, M8823) were washed three times with PBS and then blocked for 1 h in 0.5% BSA (PBS). Beads were then washed twice with IP buffer, followed by transferring of 20 µl of Flag beads into 1.0 ml of cell lysate and incubated for 2.5 h in a cold room with end-to-end rotation. Flag beads were washed twice with IP buffer, and all liquid in the tube was removed. Beads were suspended in 20 µl of 1.5× SDS-PAGE loading buffer and heated at 95 °C for 5 min. Samples were then analyzed by western blot.

### RT–qPCR

Next, 2 ml of U2OS cells was seeded into six-well plates at a density of 3 × 10^5^ cells per milliliter. When cells grew to approximately 80% confluency on the next day, cells were synchronized with 100 nM dexamethasone for 2 h. Then, dexamethasone was removed and replaced with fresh media containing 0.1% DMSO or 100 μM CCM. Cells were incubated for another 12 h and then harvested every 4 h. For unsynchronized U2OS, fresh DMEM media containing 0.1% DMSO or 100 μM CCM were added and incubated for 1 d before harvesting. Total RNA was extracted using an RNeasy Plus Mini Kit (Qiagen, 74134). Reverse transcription was performed with a High-Capacity RNA-to-cDNA Kit (Applied Biosystems, 4387406). Then, 2 μg of total RNA was used in reverse transcription. Quantitative PCR reactions were performed in a QuantStudio 7 Flex system using Fast SYBR Green reagent (Applied Biosystems, 4385612). A 10-μl reaction volume was used in a 384-well plate with at least three replicates for each reaction. Primers used here were modified based on a previous study^71^ and are listed in Supplementary Table 1. In all reactions, 300 nM forward and reverse primers were used, and β2 microglobulin (B2M) was used as an endogenous control. Relative RNA level was calculated by 2^−ΔΔCt^. Data were analyzed in GraphPad Prism 9, and circadian parameters in time-course rhythmic gene expression were determined by nonlinear regression fitting with sine wave. Wavelength was constrained to be longer than 16 h, and initial values were selected based on individual data. The goodness-of-fit (R^2^) values for each condition are as follows: 0.9101 (CRY1-DMSO), 0.9142 (CRY1-CCM), 0.9655 (CRY2-DMSO), 0.8088 (CRY2-CCM), 0.9786 (PER2-DMSO), 0.9236 (PER2-CCM), 0.8728 (PER3-DMSO) and 0.9173 (PER3-CCM). The PER1-DMSO, PER1-CCM, NR1D1-DMSO, NR1D2-CCM, CLOCK-DMSO and CLOCK-CCM data have an R^2^ value lower than 0.8.

### RIME

RIME was performed based on the protocol from Carroll et al.^53^. In summary, four 150-mm dishes of U2OS cells were cross-linked in 1% formaldehyde for each condition. Nuclear fractions were extracted according to the protocol. For each condition, 50 μl of Dynabeads Protein A (Invitrogen, 10001D) was used. Then, 4 μg of BMAL1 antibody (Abcam, ab93806) or rabbit IgG (Millipore, 12-370) was incubated with the beads at room temperature for 2 h to generate antibody-bound beads. Beads were then washed four times with 0.5% BSA to remove any unbound antibody. Lysate samples and antibody-bound beads were mixed and rotated at 4 °C overnight. Beads were washed five times with RIPA buffer and then twice with 100 mM AMBIC solution before submitting for trypsin digestion.

Peptide mixtures were obtained directly from on-bead digestion. Protein samples on beads were denatured in 8 M urea and reduced in 10 mM TCEP. Cysteines were alkylated with 50 mM 2-chloroacetamide. Protein samples were first digested using LysC (New England Biolabs (NEB), P8109S) for 2 h and then trypsin (NEB, P8101S) for overnight at 37 °C. Digestion reaction was stopped by adding formic acid to 5%. Peptide mixtures were desalted in a C18 column with 0.1% trifluoroacetic acid (TFA) as buffer A and 50% acetonitrile/0.1% TFA as buffer B. Peptides were dried in a vacuum concentrator and stored in −20 °C before injection. Samples were processed in a Bruker TIMS instrument. Raw data were analyzed in MaxQuant^72^. Proteins were identified by search against human proteomes and common contaminants. Carbamidomethylation of cystine was set as a fixed modification. False discovery rates (FDRs) were set to 1% in peptide and protein identification. Proteins were then qualified by using a standard label-free qualification strategy with at least two peptides identified in each protein. The following modifications were included in protein quantification: oxidation of methionine, acetylation of protein N-terminus, acetylation of lysine and phosphorylations of serine and threonine. The intensity of one protein was normalized to the total abundance of all proteins in each sample. The mean values of normalized intensity of the CCM-treated condition divided by that of the DMSO condition gives the fold change.

### ChIP

One 150 -mm dish of UO2S cells around 90% confluence was used for each biological replicate in ChIP studies. Cells were incubated in fresh media with 0.1% DMSO or 100 CCM for 3 h in unsynchronized condition or 24 h in synchronized U2OS cells. Cells were crosslinked for 15 min with 1% formaldehyde and then quenched with 125 mM glycine for another 5 min. Cells were then washed twice with ice-cold PBS and harvested by scraping. The chromatin complex was obtained following the RIME protocol. For each condition, 20 μl of Dynabeads Protein A (Invitrogen, 10001D) and 4 μg of BMAL1 antibody (Abcam, ab93806) or rabbit IgG (Millipore, 12-370) was used. To obtain ChIP DNA, 50 μl of 10% (w/v) Chelex 100 slurry (Bio-Rad, 1421253) was added to the final IP beads and boiled for 15 min. Then, 0.5 μl of RNase A (Invitrogen, 12091021) was added to each sample and incubated at 37 °C for 1 h on a thermal mixer (1,200 r.p.m.). Next, 1 μl of proteinase K (Invitrogen, 25530049) was added to the mixture and incubated at 65 °C for 2 h with 1,200-r.p.m. shaking. Samples were boiled for another 10 min and then centrifuged at 17,000*g* for 10 min at 4 °C. Then, 30 μl of supernatant was transferred to a new tube, and 50 μl of MilliQ water was then added to the remaining beads, vortexed and centrifuged at 17,000*g* for 10 min to provide 50 μl of additional supernatant for each sample. The ChIP DNA was further purified with DNA Clean & Concentrator (Zymo Research, D4004) for better qPCR signal. Final ChIP DNA was eluted in 20 μl of elution buffer. One microliter of ChIP DNA was used for a 10-μl qPCR reaction (SYBR Green master kit, 4385612). Three technical repeats were run in parallel for each sample to generate an *n* number of 1. Both DMSO and CCM conditions had three biological replicates (*n* = 3). The primers for ChIP–qPCR were designed in the National Center for Biotechnology Information (NCBI) with the promotor sequences of correspondent genes, and these are listed in Supplementary Table 2.

### Monitoring real-time circadian rhythms

Peritoneal macrophages were isolated from both male and female Per2–Luc mice (expressing luciferase under control of the Period 2 promoter) at age 12–19 weeks. Peritoneal exudate cells (PECs) were isolated via peritoneal lavage with ice-cold PBS. Cells were plated for 90 min and washed to remove non-adherent cells. Macrophages were then immediately treated with CCM at various concentrations or vehicle (0.1% DMSO in recording medium). Circadian oscillations were tracked in a plate reader measuring luminescence hourly across 96 h. In every experiment, three independent wells were measured from each condition. The parameters were then calculated for each well individually and averaged to give an *n* = 1 measurement of period, phase and amplitude. This experiment was then repeated on macrophages from different individual mice to increase the *n* number. Representative traces in Fig. 3e were chosen from two different biological replicates to cover the full range of concentrations analyzed. Data were first processed in MultiCycle and then smoothed in Prism (second-order smoothing, 10 neighbors). The ATP viability assay (CellTiter-Glo; Promega, G7570) was carried out on peritoneal macrophages after the 5 d of luminometry recording.

The rhythmicity of raw luminometry data was tested in BioDare2 (https://biodare2.ed.ac.uk/)^73^. No linear detrending was used. The test method selected was BD2 eJTK (the BioDare2 implementation of Empirical JTK_CYCLE with asymmetry search from Hutchison et al.^74^). The analysis presets (the set of pattern curves against which the comparison test is run) selected were eJTK Classic. Samples were included in amplitude/period/phase analysis where they were found to be rhythmic (Benjamini–Hochberg correction *P* < 0.05).

For period analysis: raw data were analyzed using default settings in BioDare2 software with the fast Fourier transform-nonlinear least squares (FFT-NLLS) algorithm^75^. Options ‘Phases by Fit’, ‘zero’ and ‘circadian’ were enabled during the process.

For phases: values were reported using a phase by fit method: one cosine function having period matching the estimated data period is fitted to the data. The fitting procedure finds phase and amplitude parameters of the cosine that follow the data the most closely and reports those values as phase and amplitude. Phase was reported relative to zero (as data are periodic and phase has been estimated, it is possible to back-calculate the data to zero). Phase was reported in circadian units (the circadian unit is 1/24 of the free running period, and it allows comparison of peaking times independently of the period).

### Protein sequence alignment

Sequences for alignment were extracted from UniProt with the following accession numbers: ARNT_P27540, ARNT2_Q9HBZ2, BMAL1_O00327 and BMAL2_Q8WYA1. Alignment was performed in Clustal X 2.1 (ref. ^76^) software or Needle^77^. The alignment graph was generated in ESPript 3.0 web service^78^.

### Thermal proteome profiling

U2OS cells overexpressing HiBiT-hBMAL1(PASB) were suspended in buffer containing 20 mM BTP pH 8.0, 150 mM NaCl and 1× cocktail protease inhibitor, and lysate was obtained after sonication and centrifugation. Cell lysate was diluted to 3 mg ml^−1^ and incubated with DMSO or 50 μM or 200 μM CCM for 1 h at room temperature. After that, a temperature gradient was applied from 37 °C to 60.9 °C for 3 min, followed by centrifugation for 30 min at the highest speed of a pre-cooled centrifuge. For each condition (DMSO or CCM treatment), supernatants across all temperatures were carefully aspirated from the tube and pooled.

Pooled samples were digested with trypsin using S-Trap Mini columns (ProtiFi) following the manufacturer’s instructions. Buffers were made using MS-grade water. In brief, SDS was added to samples containing 200 μg of protein in 100 ul to a final concentration of 5% before reduction and alkylation with 20 mM DTT and 40 mM IAA for each 30 min at room temperature. After acidification to a final concentration of 1.2% with phosphoric acid and precipitation with 7 sample volumes of binding buffer (100 mM TEAB, 90% methanol), samples were loaded step-wise on S-Trap Mini columns, washed three times with 400 μl of binding buffer and digested using 4 μg of sequencing-grade modified trypsin (Promega, V5111) in 40 μl of digestion buffer (50 mM TEAB) at 37 °C for 20 h. Peptides were eluted from column with each 40 μl of elution buffer 1 (50 mM TEAB), elution buffer 2 (0.2% formic acid) and elution buffer 3 (50% acetonitrile, 0.2% formic acid) and then dried down in a vacuum centrifuge (Genevac miVAC) overnight.

Peptides were labeled with TMT10plex mass tags (Thermo Fisher Scientific) following the manufacturer’s instructions. Dried peptides were resuspended in 102 μl of 100 mM TEAB. TMT label reagents were equilibrated at room temperature before dissolving in 42 μl of acetonitrile. The TMT10-131 label reagent was not used. Then, 100 μl of samples was mixed with 41 μl of each respective TMT label reagent on a shaker for 1 h at room temperature. The labeling was stopped by adding 8 μl of 5% hydroxylamine for 10 min at room temperature. Samples were combined and cleaned up by standard procedures using Sep-Pak Plus C18 cartridges (Waters) and then dried in a vacuum centrifuge.

Peptides were fractionated using a Dionex UltiMate 3000 system (Thermo Fisher Scientific) on a C18 column (XBridge BEH C18, 2.5 μm, 3 × 150 mm column XP, Waters) with a flow rate of 0.2 ml min^−1^. Buffer A (100% water (pH 10)) and buffer B (90% acetonitrile (pH 10)) were prepared with MS-grade water, and pH was adjusted with 17% NH_4_OH. Peptides were separated using a 100-minute gradient (0–12 min: 0.2% buffer B, 12–72 min: 2–45% buffer B, 72–80 min: 45–95% buffer B, 80–90 min at 95% buffer B, 90–92 min: 95-2% buffer B, 92–100 min at 2% buffer B). A total of 50 fractions were collected with vial switching every 2 min, which were concatenated across the gradient to yield 10 samples. Samples were dried in a vacuum centrifuge.

MS analysis was performed on an Orbitrap Fusion Lumos Tribrid mass spectrometer (Thermo Fisher Scientific) coupled to a Dionex UltiMate 3000 RSLCnano system (Thermo Fisher Scientific) via a Nanospray Flex Ion Source (Thermo Fisher Scientific) interface. Peptides were loaded onto a trap column (PepMap 100 C18, 5 μm, 5 × 0.3 mm; Thermo Fisher Scientific) at a flow rate of 10 μl min^−1^ using 0.1% TFA as loading buffer. After loading, the trap column was switched in-line with a C18 analytical column (2.0 µm particle size, 75 µm ID × 500 mm; Thermo Fisher Scientific). The column temperature was maintained at 50 °C using a PRSO-V2 column oven (Sonation). Mobile phase A consisted of 0.4% formic acid in water, and mobile phase B consisted of 0.4% formic acid in a mixture of 90% acetonitrile and 10% water. Separation was achieved using a four-step gradient over 91 min at a flow rate of 230 nl min^−1^ (increase of initial gradient from 6% to 9% solvent B within 1 min, 9% to 30% solvent B within 81 min, 30% to 65% solvent B within 8 min, 65% to 100% solvent B within 1 min and 100% solvent B for 6 min before equilibrating to 6% solvent B for 18 min before the next injection). In the liquid junction setup, electrospray ionization was enabled by applying a voltage of 1.8 kV directly to the liquid being sprayed, and a non-coated silica emitter was used. The mass spectrometer was operated in data-dependent acquisition (DDA) mode. For MS2 acquisition, we collected a 375–1,650-*m*/*z* survey scan in the Orbitrap at 120,000 resolution (FTMS1); the AGC target was set to ‘standard’; and a maximum injection time (IT) of 50 ms was applied. Intensity threshold was set to 2.5 × 10^4^; charge states 2–6 were included; and dynamic exclusion after one scan was set to 90 s. Precursor isolation was performed with quadrupole, and isolation window size was set to 0.5 *m*/*z*. The RF lens was set to 30%. MS2 was measured in the Orbitrap with a resolution of 50,000. The high-energy collision-induced dissociation (HCD) fragmentation technique was used at a fixed collision energy of 38%. The normalized AGC target was set to 200% with a maximum IT of 110 ms. Xcalibur version 4.3.73.11 and Tune 3.4.3072.18 were used to operate the instrument.

Raw MS data were converted to mzML files using the ProteoWizard msConvert tool and then searched against the human reference proteome (UP000005640; reversed protein sequences as decoys and common contaminants added, downloaded on 8 August 2023 from UniProt) using MSFragger version 3.8 (ref. ^79^), yuIonQuant version 1.9.8 (ref. ^80^) and Philosopher version 5.0.0 (ref. ^81^) within FragPipe version 20.0. The built-in ‘TMT10’ workflow was loaded with a fragment mass tolerance of 20 ppm, peptide length from 7 to 50, peptide mass from 200 Da to 5,000 Da and ‘stricttrypsin’ for in silico digestion with up to two missed cleavages. Oxidation of methionine and acetylation of protein N-termini were set as variable modifications. Carbamidomethylation of cysteine and TMT tag modification on N-termini were included as fixed modifications. All other parameters were kept as standard.

Protein intensities were further analyzed using R version 4.3.1 (https://www.R-project.org/). Proteins with missing values across all replicates and conditions and fewer than two unique peptides were removed. Abundance normalization was performed on the remaining proteins. The intensity of a protein was normalized on the total abundance of all proteins for each respective sample (labeled with a separate TMT tag). This resulted in the same total protein abundance in each sample. Next, to obtain the soluble abundance ratio per protein, the median intensity of compound-treated samples was divided by the median intensity of vehicle-treated samples and log_2_ transformed. The −log_10_(*P* value) was derived from a two-tailed Student’s *t*-test. Volcano plots were visualized using ggplot2.

### Mouse-derived macrophages

Per2–Luc mice were originally obtained from Joseph Takahashi’s laboratory (University of Texas Southwestern Medical Center) and, together with *LysM-*^*Bmal1*−/−^ mice, were maintained in the University of Oxford Biomedical Services. All protocols were approved by the University of Oxford Animal Welfare and Ethical Review Body and carried out according to the Animals (Scientific Procedures) Act 1986. Food and water were provided ad libitum, and housing was under 12-h light/dark cycles. The Cre-positive mice were age matched and sex matched with flox/flox littermate controls. BMDMs were cultured from females ages 13–17 weeks, and PECs were harvested from both male and female mice. All mice were group housed in standard housing under controlled room temperature (21–23 °C) and 50% humidity. All experimental animals were genotyped. Isolation of BMDMs was performed as follows. Tibias and femurs were dissected; bone marrow was flushed using DMEM; and the resulting single-cell suspension was plated out to non-tissue culture treated dishes. Macrophages harvested from mice at the same time are naturally synchronized.

Resulting cells were cultured in complete DMEM, P/S, 20% FBS and MCSF for 7 d. Cells were then treated with one of four conditions for 6 h before lysis for RNA extraction: DMSO (vehicle control), 100 nM LPS/DMSO, 100 μM CCM/DMSO and 100 nM LPS/100 μM CCM.

### RNA-seq studies

Cells were lysed in TRIzol reagent, and RNA was extracted using chloroform followed by RNA Clean & Concentrator (Zymo Research, R1013). RNA-seq was performed by Novogene. Messenger RNA was purified from total RNA using poly(T) oligo-attached magnetic beads. After fragmentation, the first-strand cDNA was synthesized using random hexamer primers, followed by second-strand cDNA synthesis. The library was checked with Qubit and real-time PCR for quantification and Bioanalyzer for size distribution detection. Paired-end reads were generated.

Raw data (raw reads) of fastq format were first processed using fastp software. In this step, clean data (clean reads) were obtained by removing reads containing adapter, reads containing poly(N) and low-quality reads from raw data. At the same time, Q20, Q30 and GC content for the clean data was calculated. All the downstream analyses were based on the clean data with high quality. Reference genome and gene model annotation files were downloaded from the genome website directly. Index of the reference genome was built using HISAT2 version 2.0.5, and paired-end clean 2 reads were aligned to the reference genome using HISAT2 version 2.0.5. featureCounts version 1.5.0-p3 was used to count the read numbers mapped to each gene. Then, fragments per kilobase of transcript per million mapped reads (FPKM) of each gene was calculated based on the length of the gene and reads count mapped to this gene.

Differential expression analysis was performed using the DESeq2 R package (1.20.0). The resulting *P* values were adjusted using the Benjamini–Hochberg approach for controlling the FDR. Genes with an adjusted *P* ≤ 0.05 found by DESeq2 were assigned as differentially expressed.

### Statistics and reproducibility

For RNA-seq and macrophage luminometry, sample size was determined by a power calculation based on variance from previous experiments. No statistical method was used to predetermine sample size in other experiments. Sample size was started at *n* = 3 for a minimal statistical analysis and expanded in RT–qPCR based on previous experiments. Cells and mice were randomly allocated into experimental groups. Investigators were not blinded to allocation during experiments and outcome assessment. We followed the standard criteria in data processing for proteomics and next-generation sequencing data. Proteomics data with missing values across all conditions and fewer than two peptides were removed. In RNA-seq studies, raw data were cleaned by removing reads containing adapter, reads containing poly(N) and low-quality reads from raw data. In RT–qPCR, data with extremely low reads for endogenous control (β2 microglobulin) were excluded. In Per2–Luc, extreme outliers (for example, vehicle has much lower amplitude than drug) were excluded. All experiments were repeated at least once with similar results.

### Reporting summary

Further information on research design is available in the Nature Portfolio Reporting Summary linked to this article.

## Online content

Any methods, additional references, Nature Portfolio reporting summaries, source data, extended data, supplementary information, acknowledgements, peer review information; details of author contributions and competing interests; and statements of data and code availability are available at 10.1038/s41589-025-01863-x.

## Supplementary information

Supplementary Figs. 1–6 and Supplementary Tables 1 and 2.

Reporting Summary

Supplementary Data 1 Compound screening.

## Source data

Source Data Fig. 1 Statistical source data.

Source Data Fig. 3 Statistical source data.

Source Data Fig. 3 Unprocessed western blots.

Source Data Fig. 4 Statistical source data.

Source Data Fig. 5 Statistical source data.

Source Data Fig. 5 Unprocessed western blots.

Source Data Extended Data Fig. 1 Statistical source data.

Source Data Extended Data Fig. 2 Statistical source data.

Source Data Extended Data Fig. 5 Statistical source data.

Source Data Extended Data Fig. 6 Statistical source data.

Source Data Extended Data Fig. 7 Statistical source data.

Source Data Extended Data Table 2 Statistical source data.

## Figures and Tables

**Extended Data Table 1:**
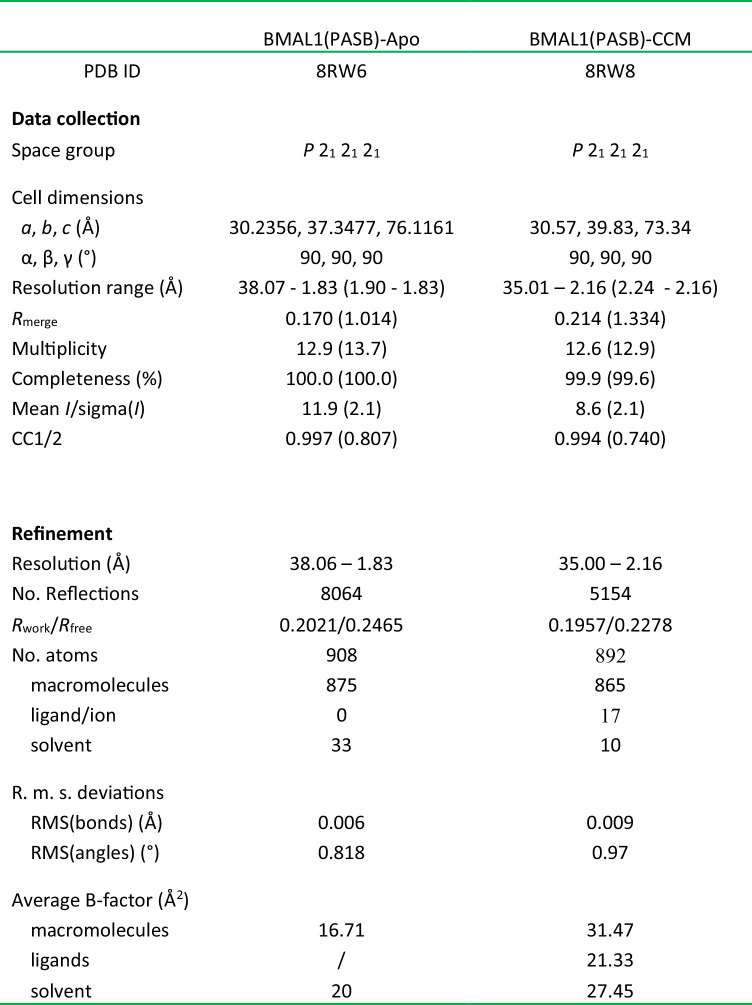
Statistics of crystal diffraction and structure refinement

Note: values in parentheses are for the highest resolution shell.

**Extended Data Table 2:**
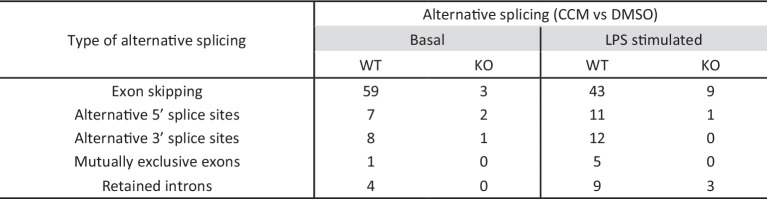
CCM increases alternative splicing in BMDMs

Note: differential splicing analysis by Novogene with RNA-seq data. Numbers (for example, 59) indicate splicing events found to be significant (FDR < 0.05) between CCM versus DMSO conditions.

## Data Availability

The structure factors and coordinates generated in this study were deposited to the RCSB Protein Data Bank (https://www.rcsb.org) with the following accession codes: 8RW6 for apo BMAL1 (PASB) and 8RW8 for BMAL1(PASB) in complex with CCM. Source data of RNA-seq were submitted to the Gene Expression Omnibus (https://www.ncbi.nlm.nih.gov/geo/*)* with accession number GSE255357. The thermal proteome profiling data were deposited to the ProteomeXchange Consortium via the PRIDE^82^ partner repository with the dataset identifier PXD049298. Source data are provided with this paper.
