# Supplementary material for: Pharmacological targeting of BMAL1 modulates circadian and immune pathways
Source: Nat Chem Biol. 2025 Mar 25;21(5):736–45. doi: 10.1038/s41589-025-01863-x (PMC12037410; doi:10.1038/s41589-025-01863-x)
Supplement: Supplementary file 1 — Supplementary Figs. 1–6 and Supplementary Tables 1 and 2. [file 41589_2025_1863_MOESM1_ESM.pdf]

nature chemical biology

Supplementary information https://doi.org/10.1038/s41589-025-01863-x

Pharmacological targeting of BMAL1 modulates circadian and immune pathways

In the format provided by the authors and unedited

Table of Contents

Supplementary Fig. 1a: 1H-NMR spectrum of CCM

Supplementary Fig. 1b: 13C-NMR spectrum of CCM

Supplementary Fig. 1c: 19F-NMR spectrum of CCM

Supplementary Fig. 1d: HPLC and MS of CCM

Supplementary Fig. 2a: 1H-NMR spectrum of A304

Supplementary Fig. 2b: 13C-NMR spectrum of A304

Supplementary Fig. 2c: HPLC and MS for A304

Supplementary Fig. 3a: 1H-NMR spectrum of A190

Supplementary Fig. 3b: 13C-NMR spectrum of A190

Supplementary Fig. 3c: HPLC and MS for A190

Supplementary Fig. 4: Synthesis routes of A190, A304 and CCM

Supplementary Fig. 5a: Replicates of the co-IP data presented in Figure 5a

Supplementary Fig. 5b:Uncropped and unprocessed blots for Supplementary Fig. 5a

Supplementary Fig. 6: Quality control of purified hBMAL1(PASB) protein

Supplementary Table 1. Primers for RT-qPCR

Supplementary Table 2. Primers for ChIP-qPCR

Supplementary Fig. 1a: 1H-NMR spectrum of CCM

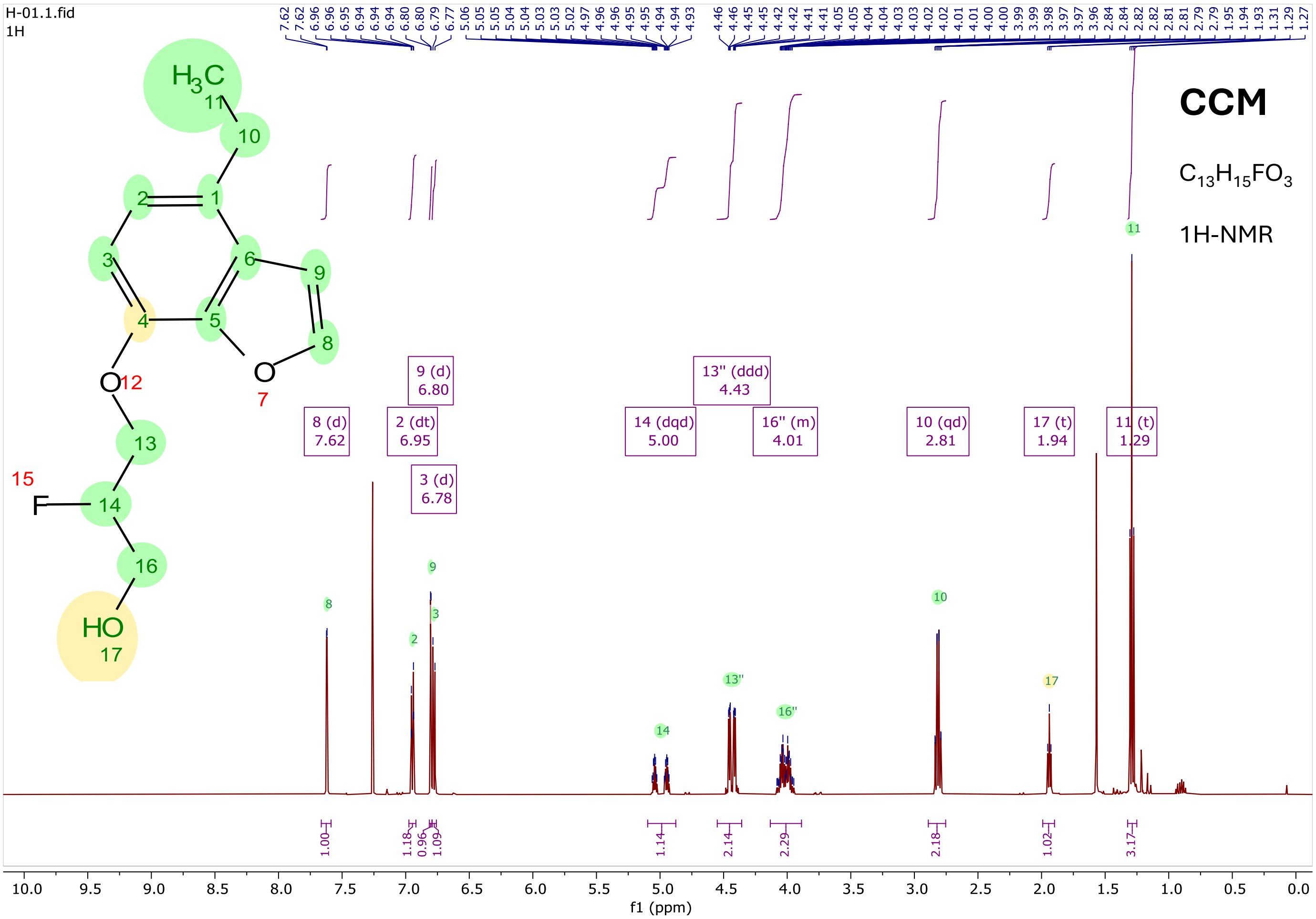

^{1}H NMR (500 MHz, $CDCl_3$) δ 7.62 (d, *J* = 2.1 Hz, 1H), 6.95 (dt, *J* = 7.9, 0.8 Hz, 1H), 6.80 (d, *J* = 2.1 Hz, 1H), 6.78 (d, *J* = 8.0 Hz, 1H), 5.00 (dqd, *J* = 47.8, 5.0, 3.7 Hz, 1H), 4.43 (ddd, *J* = 20.0, 4.9, 1.9 Hz, 2H), 4.13 – 3.89 (m, 2H), 2.81 (qd, *J* = 7.5, 0.7 Hz, 2H), 1.94 (t, *J* = 6.5 Hz, 1H), 1.29 (t, *J* = 7.6 Hz, 3H).

Supplementary Fig. 1b: 13C-NMR spectrum of CCM

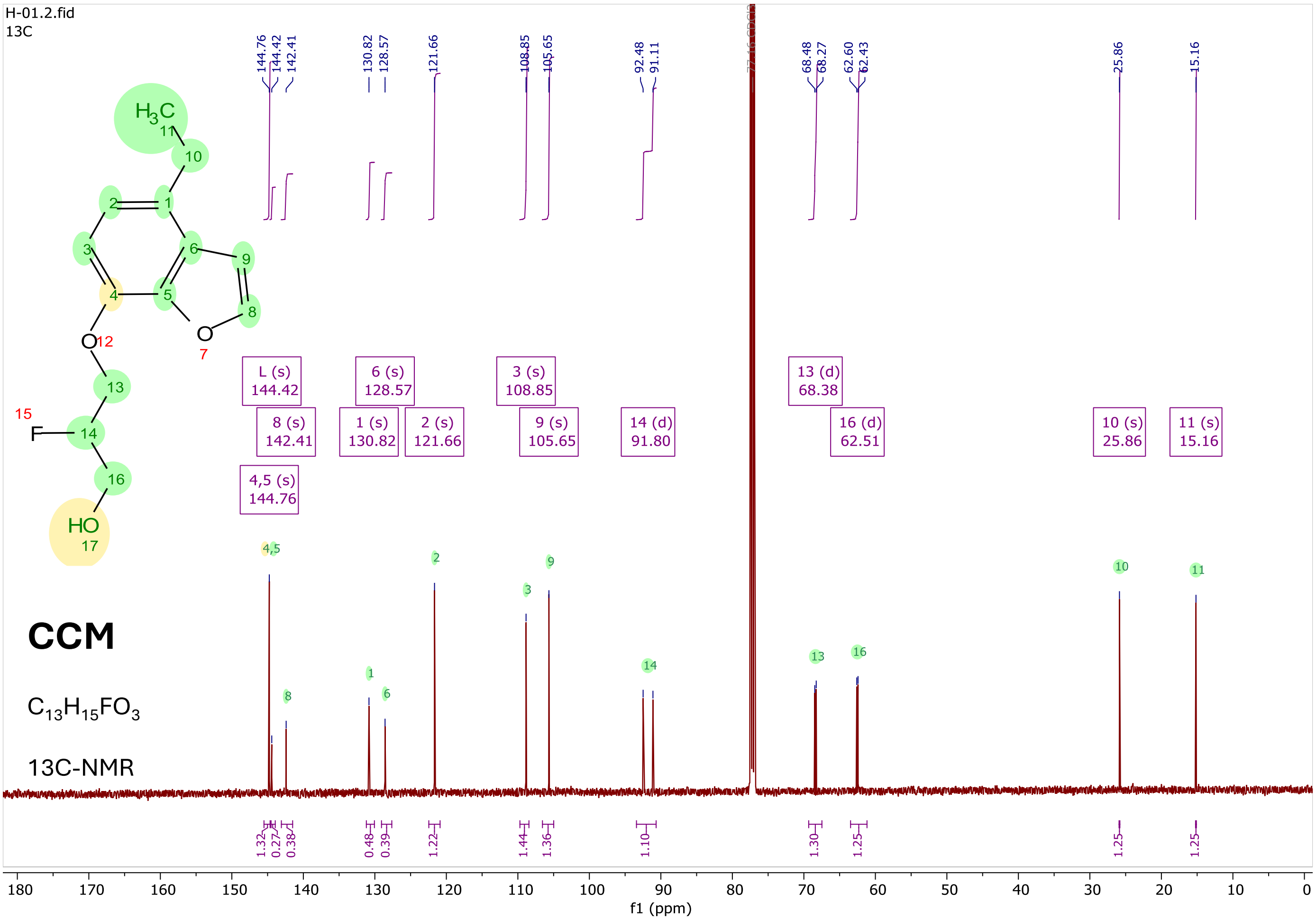

^{13}C NMR (126 MHz, $CDCl_3$) δ 144.76, 144.42, 142.41, 130.82, 128.57, 121.66, 108.85, 105.65, 91.80 (d, *J* = 173.0 Hz), 68.38 (d, *J* = 25.4 Hz), 62.51 (d, *J* = 22.2 Hz), 25.86, 15.16.

Supplementary Fig. 1c: 19F-NMR spectrum of CCM

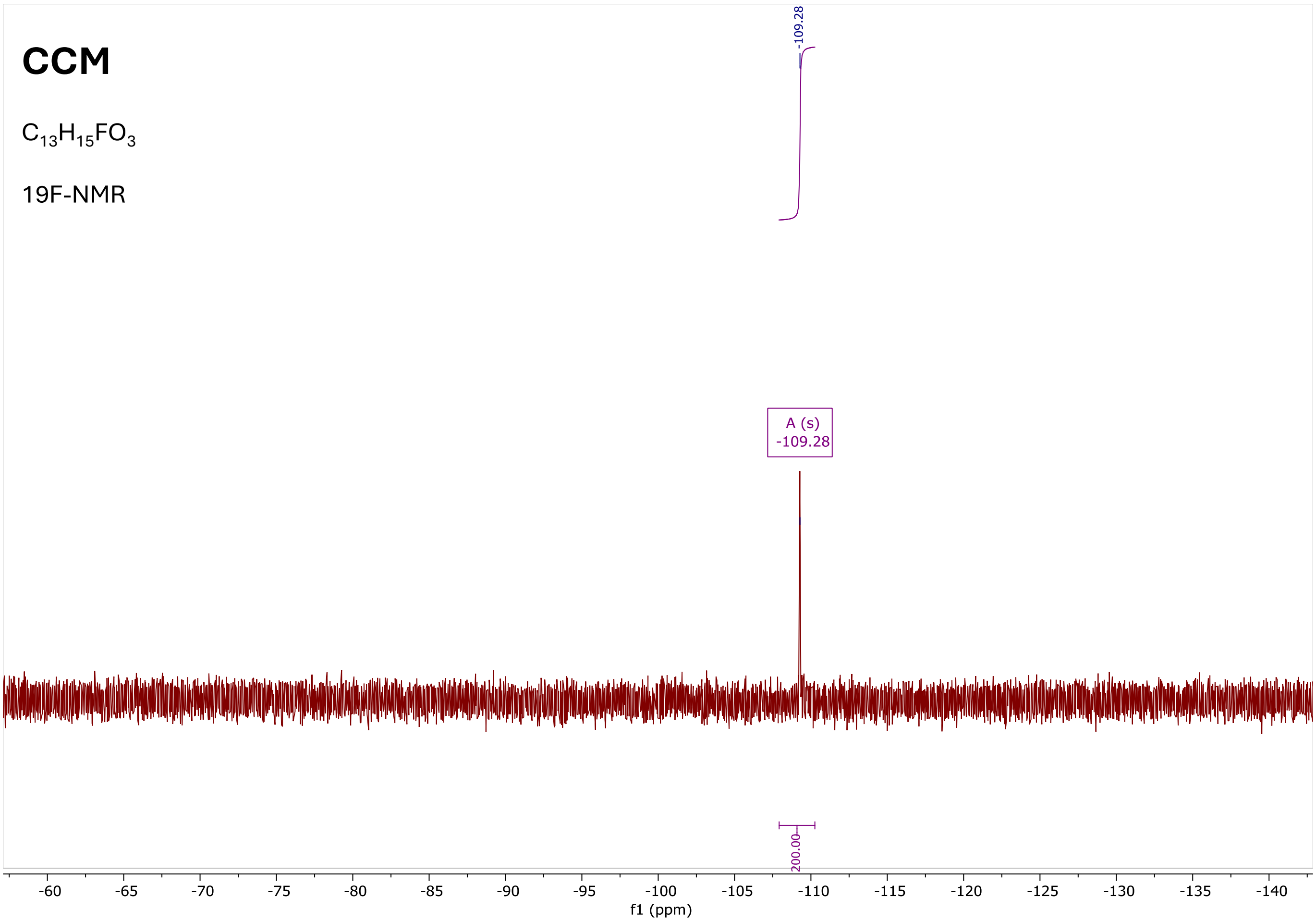

^{19}F NMR (58 MHz, $CDCl_3$) δ -109.28 (s, 1F).

Supplementary Fig. 1d: HPLC and MS of CCM

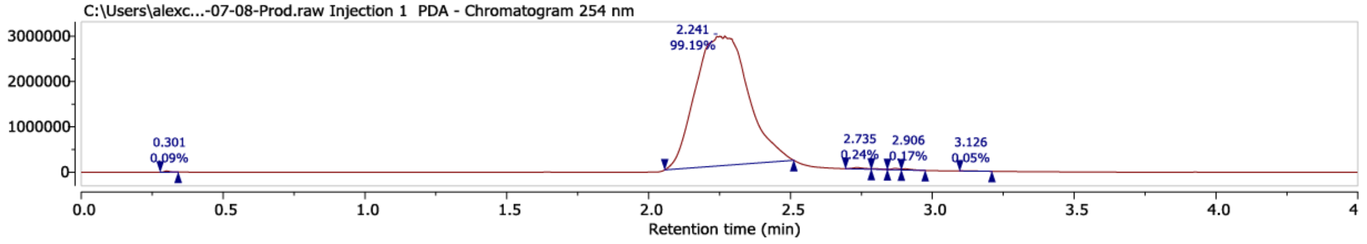

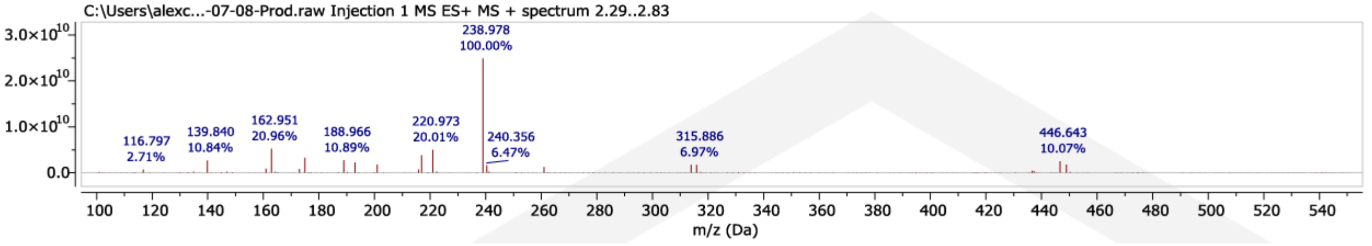

CCM

LCMS (m/z): $[M+H]^+$ calculated for $C_{13}H_{15}FO_3$, 239.1078; found, 238.978. (99.19% purity, 254 nm)

Supplementary Fig. 2a: 1H-NMR spectrum of A304

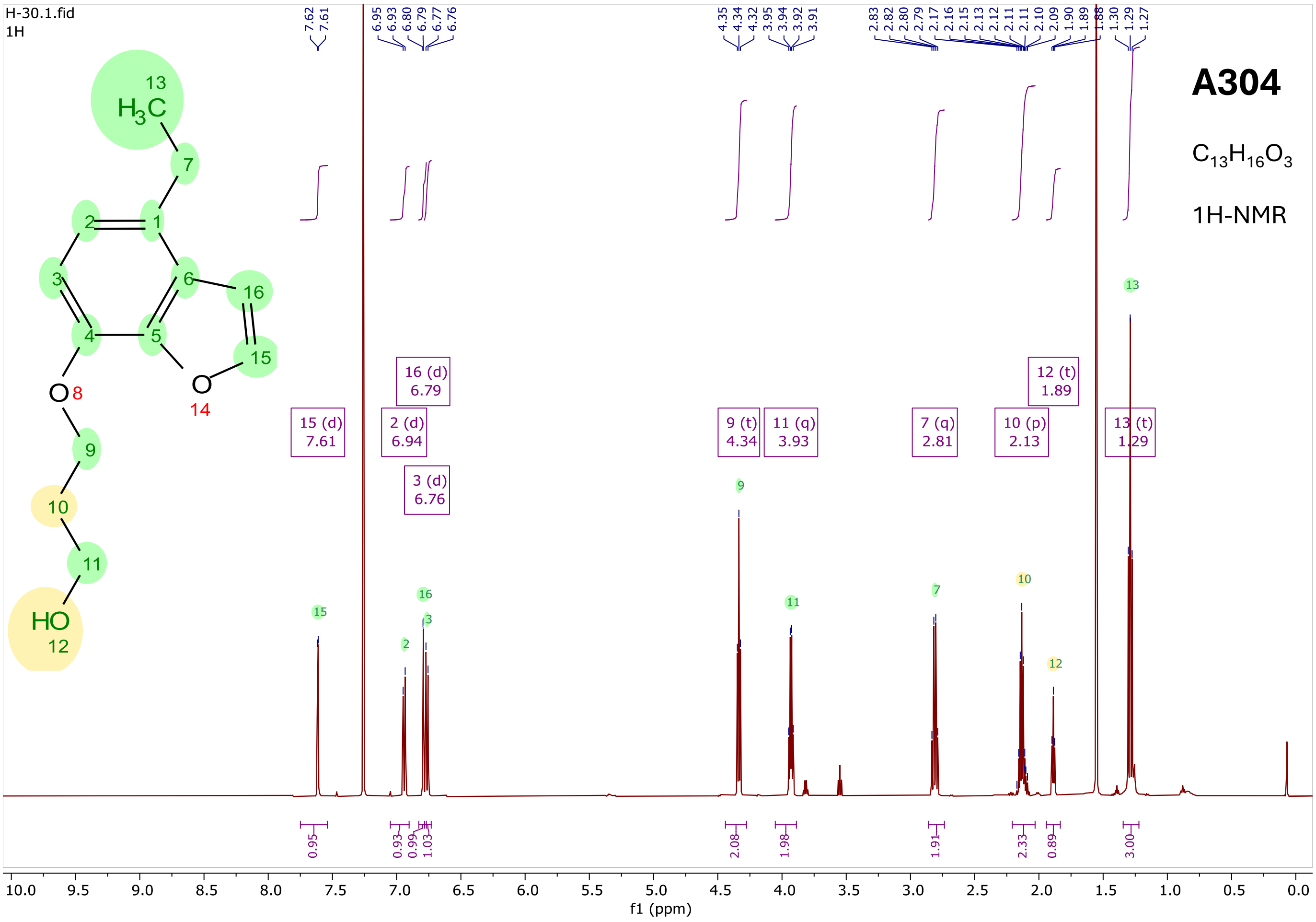

^{1}H NMR (500 MHz, $CDCl_3$) δ 7.61 (d, *J* = 2.2 Hz, 1H), 6.94 (d, *J* = 8.0 Hz, 1H), 6.79 (d, *J* = 2.2 Hz, 1H), 6.76 (d, *J* = 8.1 Hz, 1H), 4.34 (t, *J* = 6.0 Hz, 2H), 3.93 (q, *J* = 5.7 Hz, 2H), 2.81 (q, *J* = 7.6 Hz, 2H), 2.13 (p, *J* = 5.9 Hz, 2H), 1.89 (t, *J* = 5.5 Hz, 1H), 1.29 (t, *J* = 7.6 Hz, 3H).

Supplementary Fig. 2b: 13C-NMR spectrum of A304

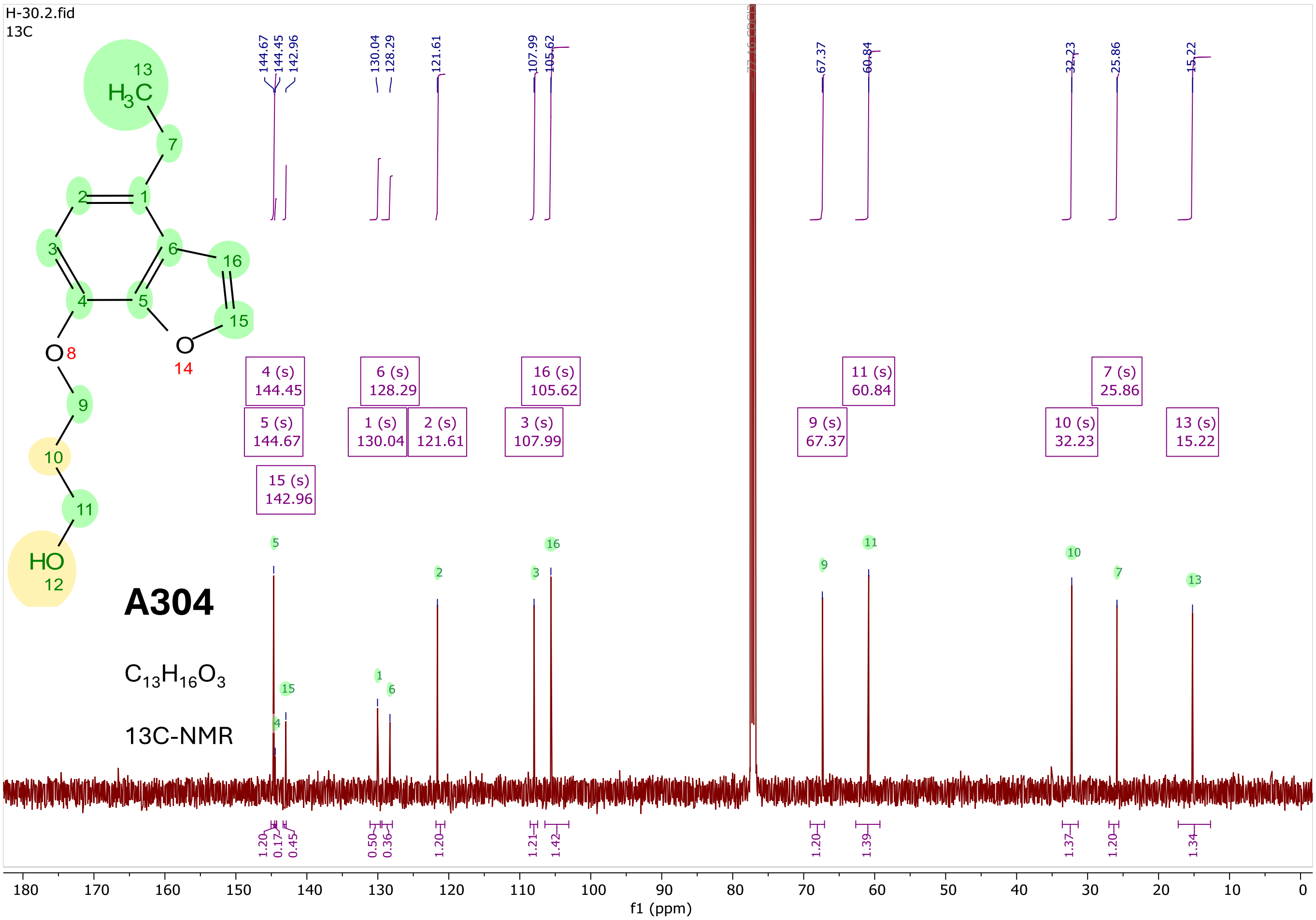

^{13}C NMR (126 MHz, $CDCl_3$) δ 144.67, 144.45, 142.96, 130.04, 128.29, 121.61, 107.99, 105.62, 67.37, 60.84, 32.23, 25.86, 15.22.

Supplementary Fig. 2c: HPLC and MS for A304

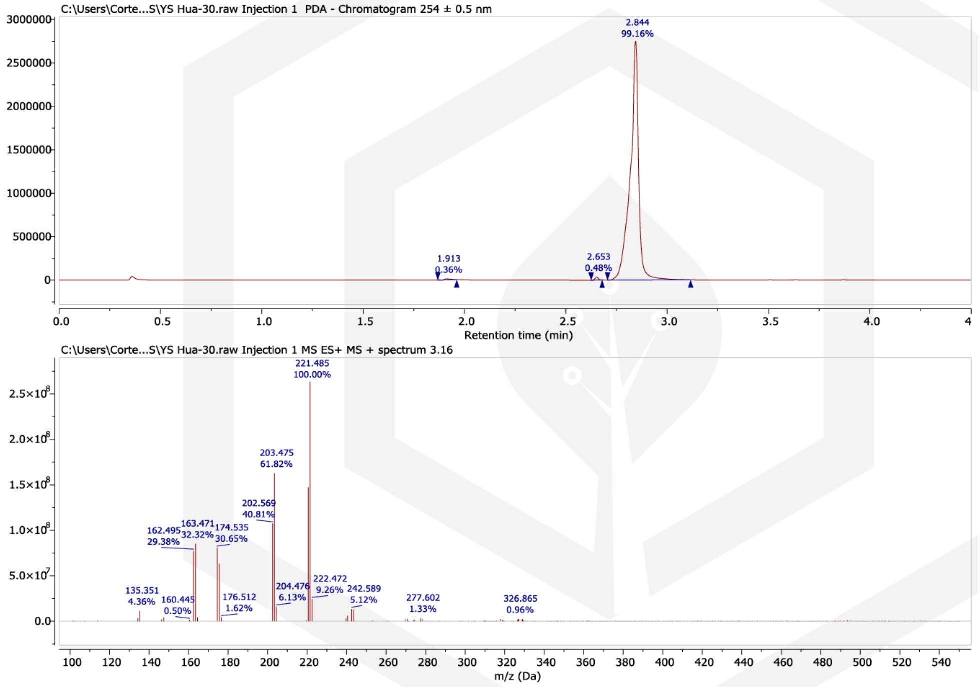

A304

LCMS (m/z): $[M+H]^+$ calculated for $C_{13}H_{16}O_3$, 221.1172; found, 221.485. (99.16% purity, 254 nm)

Supplementary Fig. 3a: 1H-NMR spectrum of A190

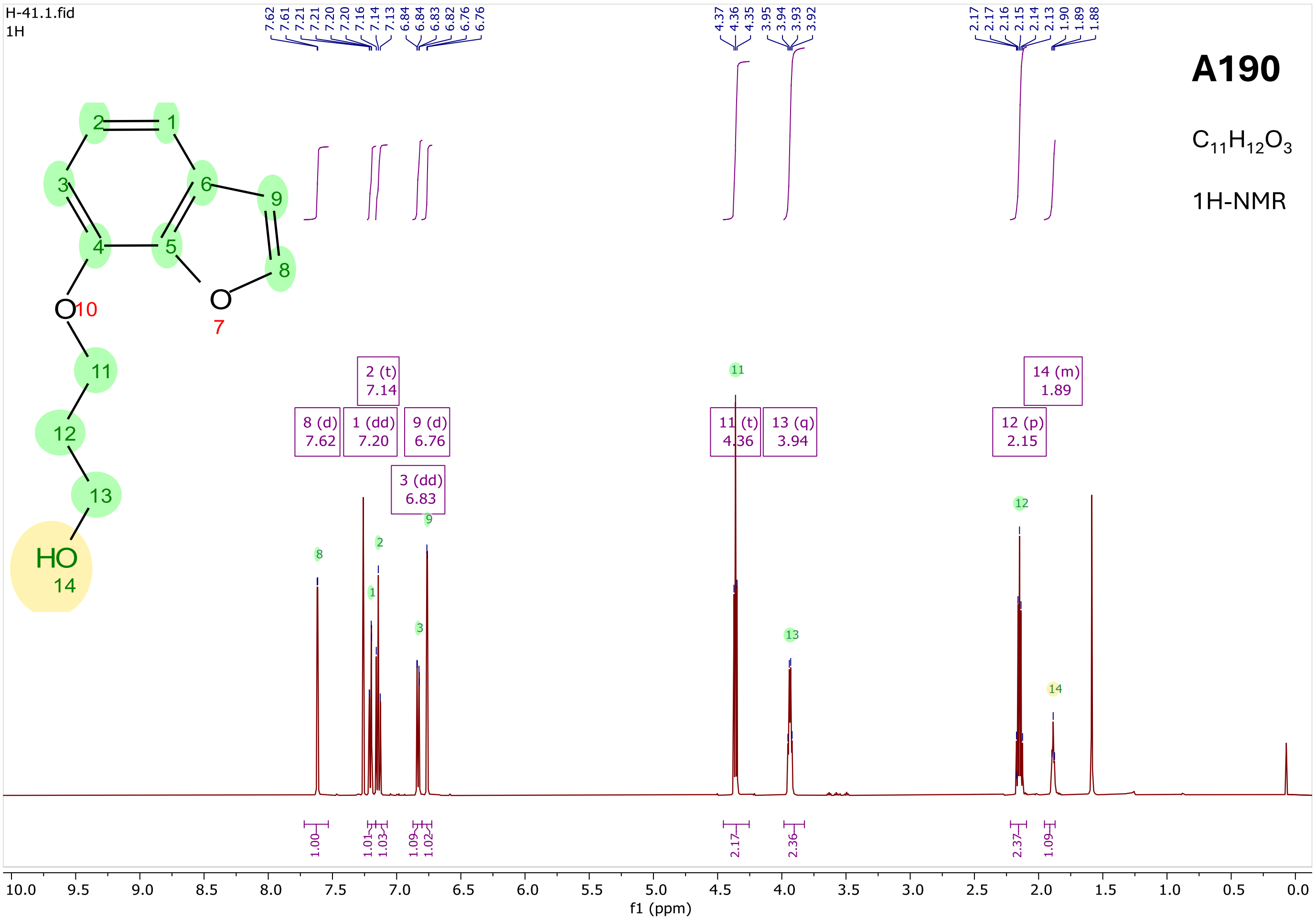

^{1}H NMR (500 MHz, $CDCl_3$) δ 7.62 (d, *J* = 2.1 Hz, 1H), 7.20 (dd, *J* = 7.8, 1.1 Hz, 1H), 7.14 (t, *J* = 7.8 Hz, 1H), 6.83 (dd, *J* = 7.9, 1.1 Hz, 1H), 6.76 (d, *J* = 2.1 Hz, 1H), 4.36 (t, *J* = 6.0 Hz, 2H), 3.94 (q, *J* = 5.4 Hz, 2H), 2.15 (p, *J* = 5.9 Hz, 2H), 1.95 – 1.87 (m, 1H).

Supplementary Fig. 3b: 13C-NMR spectrum of A190

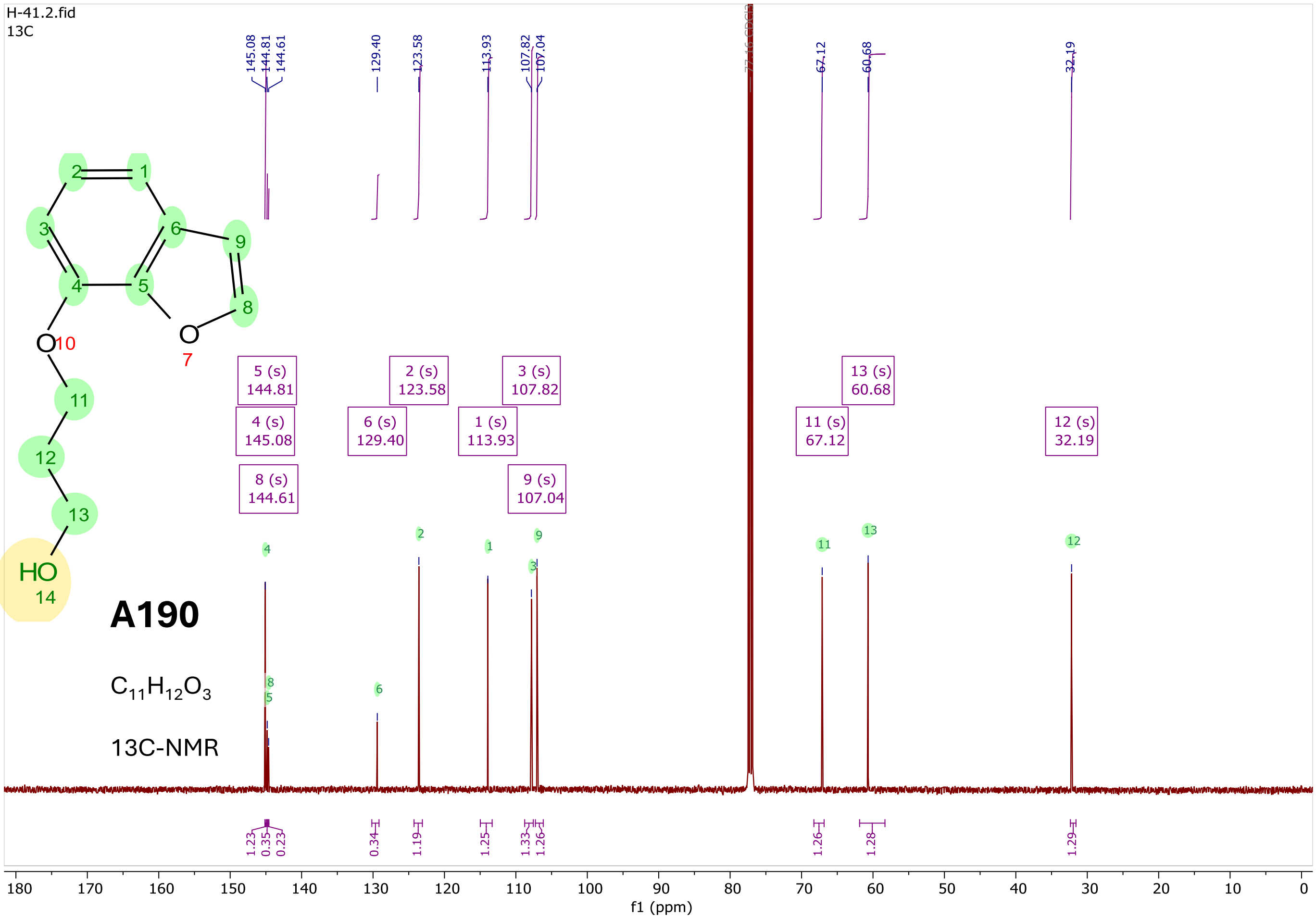

^{13}C NMR (126 MHz, $CDCl_3$) δ 145.08, 144.81, 144.61, 129.40, 123.58, 113.93, 107.82, 107.04, 67.12, 60.68, 32.19.

Supplementary Fig. 3c: HPLC and MS for A190

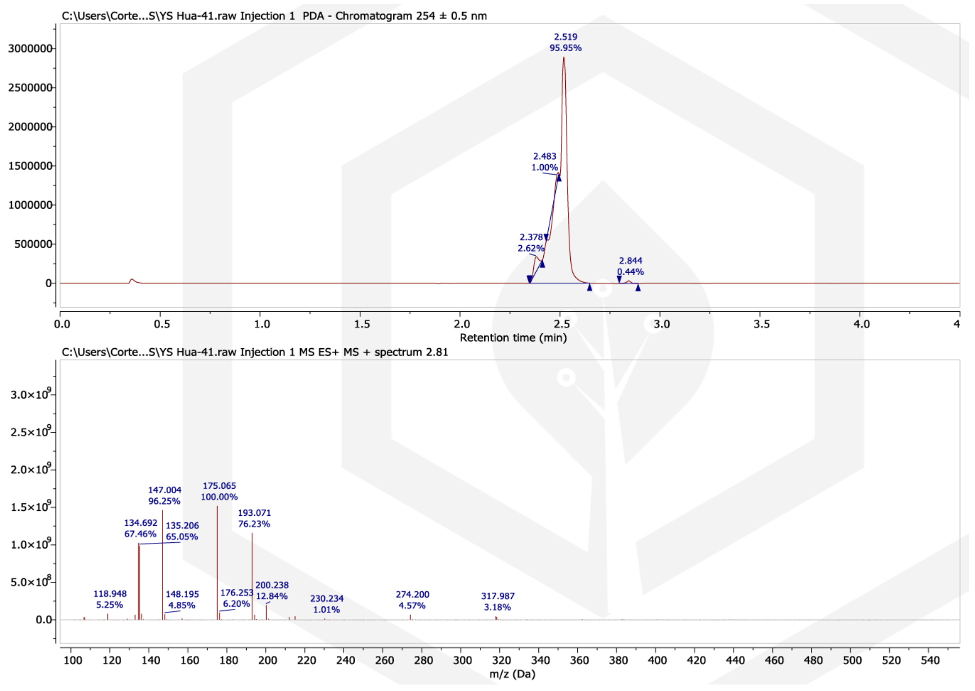

A190

LCMS (m/z): $[M+H]^+$ calculated for $C_{11}H_{12}O_3$, 193.0859; found, 193.071. (95.95% purity, 254 nm)

Supplementary Fig. 4: Synthesis routes of A190 (**a**), A304 (**b**) and CCM (**c**). Compounds were designed in Maestro (Schrodinger) and synthesized by Cortex Organics Ltd., Oxford UK.

a

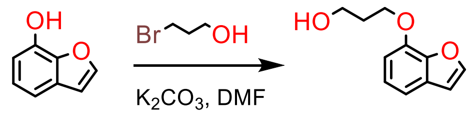

A190

b

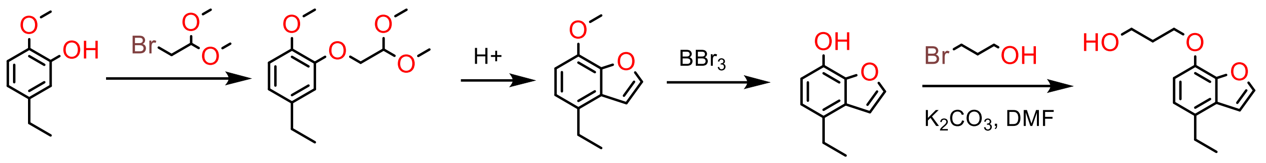

A304

c

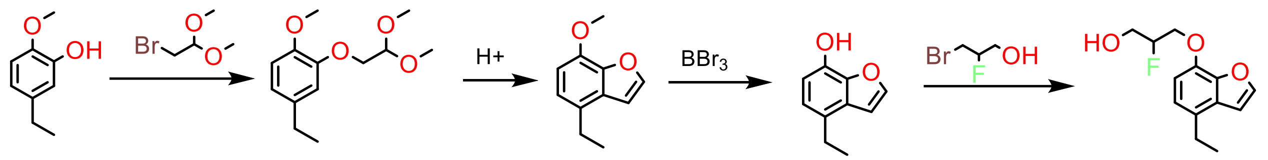

CCM

Supplementary Fig. 5a: Replicates of the co-IP data presented in Figure 5a. The red arrow indicates a transfer issue.

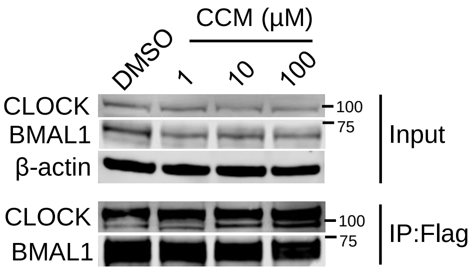

Repeat 01

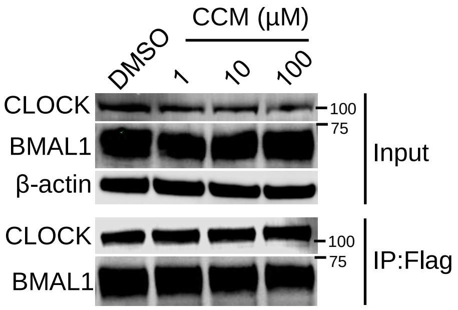

Repeat 02

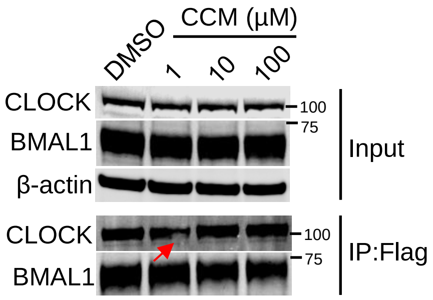

Repeat 03

Supplementary Fig. 5b:uncropped and unprocessed blots for Supplementary Fig. 5a.

anti-BMAL1
anti-CLOCK

Repeat_01

Stripping and reprobing
anti-β-actin

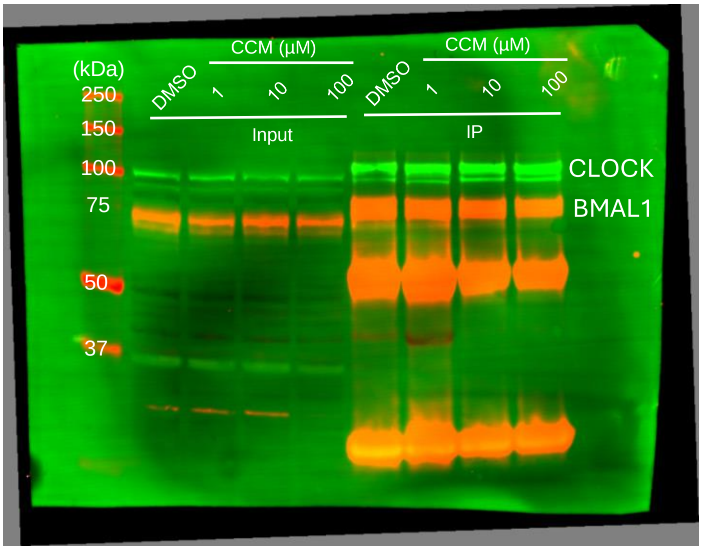

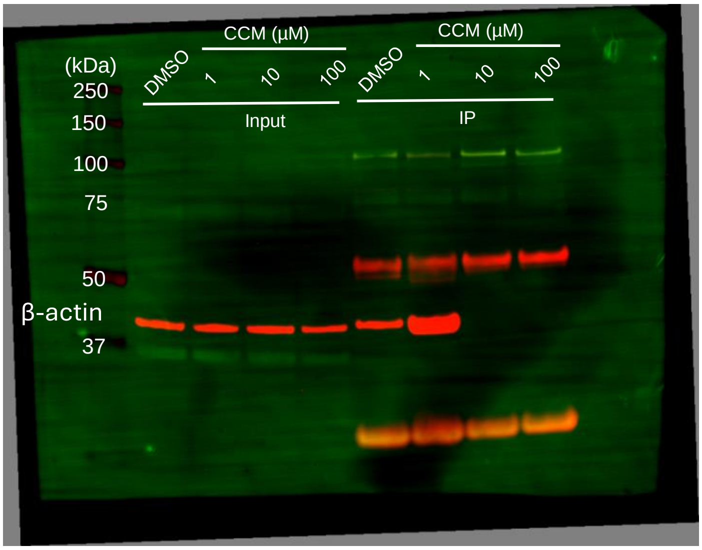

Repeat_03

anti-BMAL1
anti-CLOCK
anti-β-actin

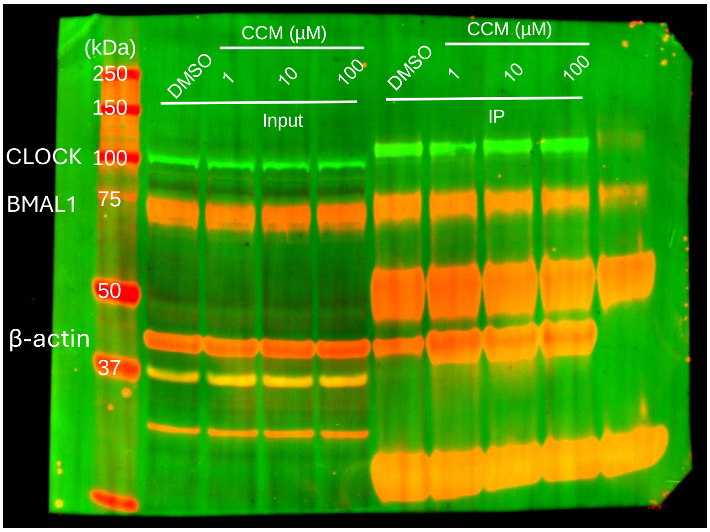

Supplementary Fig. 6: Quality control of purified hBMAL1(PASB) protein, based on SDS-PAGE and MS.

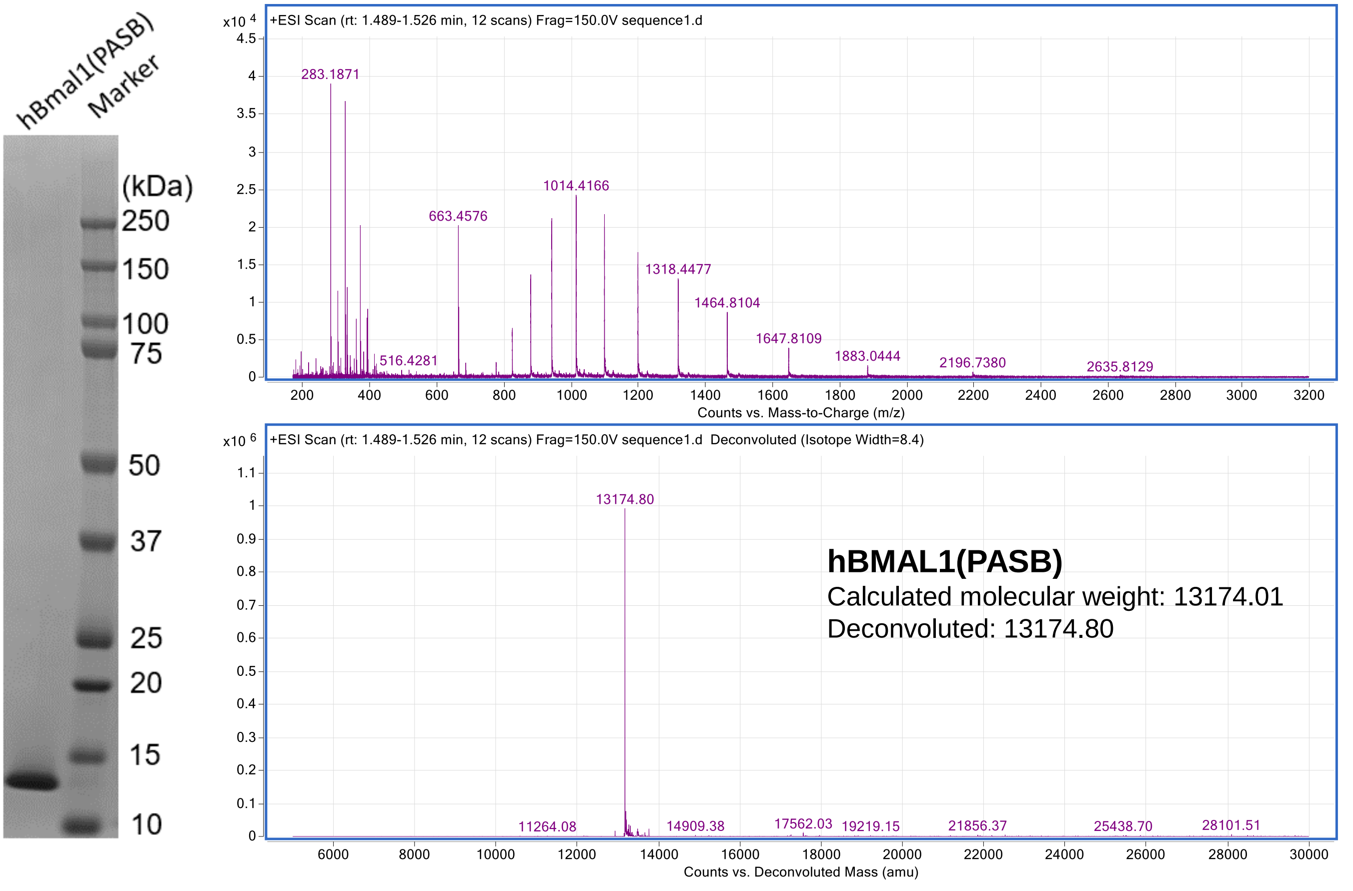

Supplementary Table 1. Primers for RT-qPCR

Name	Sequence (5’ to 3')
BMAL2-rtq-F	ACTTGGTGCTGGTAGTATTGGA
BMAL2-rtq-R	TGTTGGACTCGAATCATCAAGG
NPAS2-rtq-F	CAGGACTGGAAGCCATCATT
NPAS2-rtq-R	GATGCTGCCGTCTGTTGTC
B2M-rtq-F	CTACACTGAATTCACCCCCACTG
B2M-rtq-R	ACCTCCATGATGCTGCTTACATG
CLOCK-rtq-F	GTAGCTTGTGGGGCAGTCAT
CLOCK-rtq-R	TGGAGCAACCTAGAAGTCTGT
BMAL1-rtq-F	ATTCTTGGTGAGAACCCCCAC
BMAL1-rtq-R	TGTAGTGTTTACAGCGGCCA
PER1-rtq-F	AGGATCCCATTTGGCTGCTC
PER1-rtq-R	TCCACACAGGCCATCACAT
PER2-rtq-F	AGCTGCTTGGACAGCGTCATCA
PER2-rtq-R)	CCTTCCGCTTATCACTGGACCT
PER3-rtq-F	AGACACCTGAGCGCATTCTC
PER3-rtq-R	GTGACACAGGCTTGAATGTCG
CRY1-rtq-F	GCAGTTGCTTGCTTCCTGAC
CRY1-rtq-R	GACAGGCAAATAACGCCTGA
CRY2-rtq-F	CCTGAGACTGCAGAGCCCTT
CRY2-rtq-R	CTGGCGTGCTACAGGTACTC
NR1D1-rtq-F	CTTTGAGGTGCTGATGGTGCG
NR1D1-rtq-R	CACCGAAGCGGAATTCTCCA
NR1D2-rtq-F	GGAGGAAGAATGCATCTGGTTTG
NR1D2-rtq-R	GAACCCAGGAATACGCTTTGC
RORA-rtq-F	AGCAGATCGCTCATGGCTG
RORA-rtq-R	GAAGTCGCACAATGTCTGGG
RORB-rtq-F	CTGATATCTCCAGACCGAGCC
RORB-rtq-R	CAAACTGCCGTGATGGTTGG
RORC-rtq-F	GAAGTGACTGGCTACCAGAGG
RORC-rtq-R	CACTTCCATTGCTCCTGCTTTG

Supplementary Table 2. Primers for ChIP-qPCR

Name	Sequence (5' to 3')
PER1_ChIP_F	ACGCTTATTGGCTTAGGGGC
PER1_ChIP_R	AATGAGAAGACGATGGCGGC
PER2_ChIP_F	CGCCCGTCGCTCTTTTTACATA
PER2_ChIP_R	CGTCTCCATTGAGGAACCGAC
PER3_ChIP_F	GCTTATATAACCCGGGCGCA
PER3_ChIP_R	GTGCGATTGGTCGTCGGG
CRY1_ChIP_F	AACTTGTTGACCCTCGTCCA
CRY1_ChIP_R	AACGCGATTTGCTTCCAAGG
CRY2_ChIP_F	CGTGGGTAAGAGATCCGCTG
CRY2_ChIP_R	CCCCCTCACGTTCCTACCAT
