# Supplementary material for: Pharmacological targeting of BMAL1 modulates circadian and immune pathways
Source: Nat Chem Biol. 2025 Mar 25;21(5):736–45. doi: 10.1038/s41589-025-01863-x (PMC12037410; doi:10.1038/s41589-025-01863-x)
Supplement: Supplementary file 2 — Reporting Summary [file 41589_2025_1863_MOESM2_ESM.pdf]

nature portfolio

Corresponding author(s): Fraydoon Rastinejad

Last updated by author(s): Feb 4, 2025

Reporting Summary

Nature Portfolio wishes to improve the reproducibility of the work that we publish. This form provides structure for consistency and transparency in reporting. For further information on Nature Portfolio policies, see our Editorial Policies and the Editorial Policy Checklist.

Statistics

For all statistical analyses, confirm that the following items are present in the figure legend, table legend, main text, or Methods section.

n/a	Confirmed	
☐	☒	The exact sample size (*n*) for each experimental group/condition, given as a discrete number and unit of measurement
☐	☒	A statement on whether measurements were taken from distinct samples or whether the same sample was measured repeatedly
☐	☒	The statistical test(s) used AND whether they are one- or two-sided<br>*Only common tests should be described solely by name; describe more complex techniques in the Methods section.*
☒	☐	A description of all covariates tested
☐	☒	A description of any assumptions or corrections, such as tests of normality and adjustment for multiple comparisons
☐	☒	A full description of the statistical parameters including central tendency (e.g. means) or other basic estimates (e.g. regression coefficient) AND variation (e.g. standard deviation) or associated estimates of uncertainty (e.g. confidence intervals)
☐	☒	For null hypothesis testing, the test statistic (e.g. F, t, r) with confidence intervals, effect sizes, degrees of freedom and P value noted<br>*Give P values as exact values whenever suitable.*
☒	☐	For Bayesian analysis, information on the choice of priors and Markov chain Monte Carlo settings
☒	☐	For hierarchical and complex designs, identification of the appropriate level for tests and full reporting of outcomes
☒	☐	Estimates of effect sizes (e.g. Cohen's *d*, Pearson's *r*), indicating how they were calculated

Our web collection on statistics for biologists contains articles on many of the points above.

Software and code

Policy information about availability of computer code

Data collection	UNICORN start v1.1, PHERAstar MARS v3.32, QuantStudio Real-Time PCR v7, Nano ITCRun v3.6.5.0, Biacor S200 Control v1.1, Image Studio v3.1
Data analysis	Graph Pad Prism 10, Excel, PyMOL v2.3.0, PyVOL 1.7.6, Image Studio v 5.2, NanoAnalyze v3.10.0, Protein thermal shift software v1.4, Biacore S200 Evaluation v1.1.1, Biodare2, R v.4.3.1 , MSFragger v.3.8, yuIonQuant v.1.9.8 , Philosopher v.5.0.0 , Fragpipe v.20.0, DESeq2 R package (1.20.0), Hisat2 v2.0.5, WinCoot v0.7, Phenix v1.18.2, CCP4i2, MaxQuant v2.3.1.0, DataWarrior V6.1.0, MestreNova v15, Schrodinger Suites 2021-03, ESPript 3.0, ClustalX 2.1, featureCounts v1.5.0

For manuscripts utilizing custom algorithms or software that are central to the research but not yet described in published literature, software must be made available to editors and reviewers. We strongly encourage code deposition in a community repository (e.g. GitHub). See the Nature Portfolio guidelines for submitting code & software for further information.

Data

Policy information about availability of data

All manuscripts must include a data availability statement. This statement should provide the following information, where applicable:

- Accession codes, unique identifiers, or web links for publicly available datasets
- A description of any restrictions on data availability
- For clinical datasets or third party data, please ensure that the statement adheres to our policy

The structure factors and coordinates generated in this study were deposited to RCSB Protein Data Bank (https://www.rcsb.org) with following accession codes: 8RW6 (apo BMAL1 PAS-B) and 8RW8 (BMAL1 PAS-B in complex with CCM). Source data of RNA-seq was submitted into Gene Expression Omnibus (https://www.ncbi.nlm.nih.gov/geo/) with GEO accession GSE255357. The mass spectrometry proteomics data have been deposited to the ProteomeXchange Consortium via the PRIDE 63 partner repository with the dataset identifier PXD049298.
Other publicly available data from previous publications can be found in RCSB Protein Data Bank with PDB ID 4F3L, 4H10, 6E3S, 2KDK, 4ZP4 and 7XI3.

Research involving human participants, their data, or biological material

Policy information about studies with human participants or human data. See also policy information about sex, gender (identity/presentation), and sexual orientation and race, ethnicity and racism.

Reporting on sex and gender	Not applicable.
Reporting on race, ethnicity, or other socially relevant groupings	Not applicable.
Population characteristics	Not applicable.
Recruitment	Not applicable.
Ethics oversight	Not applicable.

Note that full information on the approval of the study protocol must also be provided in the manuscript.

Field-specific reporting

Please select the one below that is the best fit for your research. If you are not sure, read the appropriate sections before making your selection.

☒ Life sciences ☐ Behavioural & social sciences ☐ Ecological, evolutionary & environmental sciences

For a reference copy of the document with all sections, see nature.com/documents/nr-reporting-summary-flat.pdf

Life sciences study design

All studies must disclose on these points even when the disclosure is negative.

Sample size	For RNA-seq and real-time circadian rhythm, sample size was determined by a power calculation based on variance from previous experiments. No statistical method was used to predetermine sample size in other experiments. Sample size was started at n=3 for a minimal statistical analysis, and expanded in RT-qPCR based on previous experiments.
Data exclusions	In thermal proteome profiling, proteins with missing values across all replicates and conditions and less than two unique peptides were removed. In RNA-seq studies, raw data was cleaned by removing reads containing adapter, reads containing poly-N and low-quality reads from raw data. In RIME study, proteins quantified to zero in all conditions were excluded. In Per2-Luc, extreme outliers (e.g vehicle has much lower amplitude than drug) were excluded. In RT-qPCR, data with extremely low read for endogenous control (β2 microglobulin) was excluded.
Replication	For statistical analysis, sample sizes are indicated in the related text, and we used a minimum of n=3 per group.<br>For others including crystallization, all experiments have been repeated at least once with similar results. An attempt for protein crystallization was not successful, which may be caused by the difference between the temperature for plate setup (20°C) and incubation (4°C). Not all attempts for RT-qPCR were successful, which should relate to the confluence of cells and sample size.
Randomization	Cells and mice were randomly allocated into experimental groups.
Blinding	Investigators were not blinded to allocation during experiments and outcome assessment.

Reporting for specific materials, systems and methods

We require information from authors about some types of materials, experimental systems and methods used in many studies. Here, indicate whether each material, system or method listed is relevant to your study. If you are not sure if a list item applies to your research, read the appropriate section before selecting a response.

Materials & experimental systems

n/a	Involved in the study
☐	☒ Antibodies
☐	☒ Eukaryotic cell lines
☒	☐ Palaeontology and archaeology
☐	☒ Animals and other organisms
☒	☐ Clinical data
☒	☐ Dual use research of concern
☒	☐ Plants

Methods

n/a	Involved in the study
☒	☐ ChIP-seq
☒	☐ Flow cytometry
☒	☐ MRI-based neuroimaging

Antibodies

Antibodies used

Primary antibodies: BMAL1 (Santa Cruz Biotechnology, sc-365645x, 1:1000 dilution), CLOCK (Cell signaling technology, 5157S, 1:1000 dilution), alpha Tubulin (Abcam, ab4074, 1:5000 dilution), Lamin B1 (Santa Cruz Biotechnology, sc-374015, 1:200 dilution), beta Actin (Abcam, ab184092, 1:10000 dilution), Myc-tag antibody (Cell signaling technology, 2278S, 1:1000 dilution), Flag-tag antibody (Sigma, F4042, 1:1000 dilution).
Secondary antibody :anti-Mouse (LI-COR, 926-68070, 1:15000 dilution), anti-Rabbit (LI-COR, 926-32211, 1:15000 dilution)

Validation

Bmal1 and Clock antibodies were validated in Western blot and siRNA knock down experiments.

Other antibodies were validated by manufacturers and relevant publications.
alpha Tubulin: Human; 1 µg/mL; Can be blocked with alpha Tubulin peptide (ab23537).
Lamin B1: Lamin B1 Antibody (B-10): sc-374015. Western blot analysis of Lamin B1 expression in HeLa whole cell lysates.
beta Actin: Mouse Monoclonal beta Actin antibody - conjugated to Alexa Fluor® 680. Suitable for WB and reacts with Mouse, Human samples.
Myc-tag antibody: Myc-Tag (71D10) Rabbit mAb detects exogenously expressed Myc-tagged proteins in cells expressed under a CMV promoter.
Flag-tag antibody: Anti-FLAG M5 monoclonal antibody detects an N-terminal Met-FLAG fusion protein in a crude extract of mammalian cells with minimal cross reactivity. Anti-FLAG M5 monoclonal antibody detects less than 1 ng of Met-FLAG-BAP fusion protein on a dot blot using chemiluminescent detection.

Eukaryotic cell lines

Policy information about cell lines and Sex and Gender in Research

Cell line source(s)

HEK293T (ATCC, CRL-3216) isolated from the kidney of a patient.
U2OS (ATCC, HTB-96) from a moderately differentiated sarcoma of the tibia of a 15-year-old, white, female osteosarcoma patient.
We prepared the mouse derived macrophages (both peritoneal and bone marrow derived) for this study. Peritoneal macrophage (Per2Luc): 12-19 weeks, male and female. BMDM (LysM Cre, Bmal1-flox): 13-17 weeks, female.

Authentication

HEK293T and U2OS purchased from ATCC (STR profiling as quality control). Macrophages were verified by examination of cell morphology under microscope and luminescence signal.

Mycoplasma contamination

Not tested.

Commonly misidentified lines
(See ICLAC register)

None

Animals and other research organisms

Policy information about studies involving animals; ARRIVE guidelines recommended for reporting animal research, and Sex and Gender in Research

Laboratory animals

LysM-Bmal1-/- mice and Per2-Luc mice were maintained in the University of Oxford Biomedical Services.
Peritoneal macrophage (Per2Luc): 12-19 weeks, male and female. BMDM (LysM Cre, Bmal1-flox): 13-17 weeks, female

Wild animals

No wild animals were used.

Reporting on sex

BMDMs were cultured from females ages 13-17 weeks, peritoneal exudate cells (PECs) were harvested from both male and female mice

Field-collected samples

No field collected samples.

Ethics oversight	All protocols were approved by the University of Oxford Animal Welfare and Ethical Review Body and carried out according to the Animals (Scientific Procedures) Act 1986.

Note that full information on the approval of the study protocol must also be provided in the manuscript.

Plants

Seed stocks	Not applicable.
Novel plant genotypes	Not applicable.
Authentication	Not applicable.
