# Supplementary material for: Pharmacological targeting of BMAL1 modulates circadian and immune pathways
Source: Nat Chem Biol. 2025 Mar 25;21(5):736–45. doi: 10.1038/s41589-025-01863-x (PMC12037410; doi:10.1038/s41589-025-01863-x)

uncropped and unprocessed blots for Fig.3d

anti-BMAL1
anti-β-actin

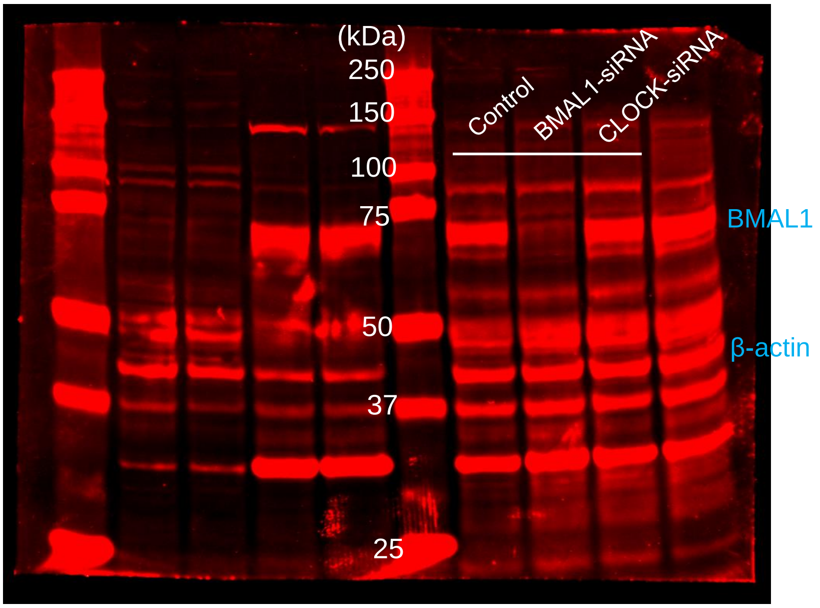

Stripping and reprobing

anti-ClLOCK

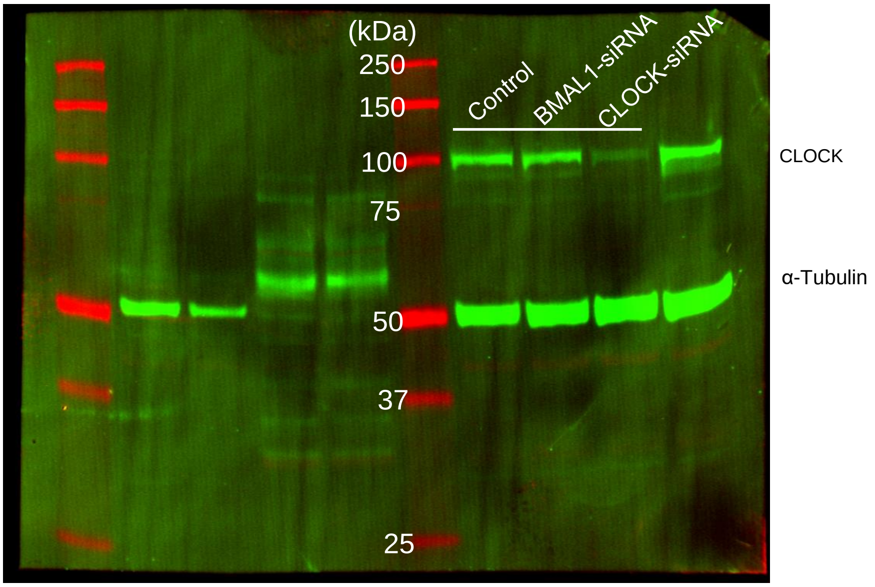

Supplement: Supplementary file 6 — Unprocessed western blots. [file 41589_2025_1863_MOESM6_ESM.pdf]
