# Supplementary material for: Pharmacological targeting of BMAL1 modulates circadian and immune pathways
Source: Nat Chem Biol. 2025 Mar 25;21(5):736–45. doi: 10.1038/s41589-025-01863-x (PMC12037410; doi:10.1038/s41589-025-01863-x)
Supplement: Supplementary file 15 — Statistical source data. [file 41589_2025_1863_MOESM15_ESM.zip › Alternative splicing - Novogene analysis/AS.README.pdf]

AS

1 Introduction

Alternative splicing analysis was performed by the software rMATS, a statistical method for robust and flexible detection of differential AS from replicate RNA-Seq data. It identifies alternative splicing events corresponding to all major types of alternative splicing patterns and calculates the *P* value and FDR for differential splicing. These types include exon skipping (SE), alternative 5′ splice sites (A5SS), alternative 3′ splice sites (A3SS), mutually exclusive exons (MXE), and retained introns (RI).

2 Directory tree

1.daslist/ TreatvsControl/

|-- TreatvsControl_SE.xls [SE event differential alternative splicing analysis result]

|-- TreatvsControl_RI.xls [RI event differential alternative splicing analysis result]

|-- TreatvsControl_A5SS.xls [A5SS event differential alternative splicing analysis result]

|-- TreatvsControl_A3SS.xls [A3SS event differential alternative splicing analysis result]

|-- TreatvsControl_MXE.xls [MXE event differential alternative splicing analysis result]

|-- TreatvsControl_SE_significant.xls [SE event significantly differential alternative splicing analysis result]

|-- TreatvsControl_RI_significant.xls [RI event significantly differential alternative splicing analysis result]

|-- TreatvsControl_A5SS_significant.xls [A5SS event significantly differential alternative splicing analysis result]

|-- TreatvsControl_A3SS_significant.xls [A3SS event significantly differential alternative splicing analysis result]

|-- TreatvsControl_MXE_significant.xls [MXE event significantly differential alternative splicing analysis result]

2. dasplot/ TreatvsControl/

|-- SE

|-- Event_name.png [plot of SE results , png format]

|-- Event_name.pdf [plot of SE results, pdf format]

|-- RI

|-- Event_name.png [plot of RI results , png format]

|-- Event_name.pdf [plot of RI results , pdf format]

|-- A5SS

|-- Event_name.png [plot of A5SS results , png format]

|-- Event_name.pdf [plot of A5SS results , pdf format]

|-- A3SS

|-- Event_name.png [plot of A3SS results , png format]

|-- Event_name.pdf [plot of A3SS results , pdf format]

|-- MXE

|-- Event_name.png [plot of MXE results , png format]

|-- Event_name.pdf [plot of MXE results , pdf format]

3 File Formats Specification

3.1 TreatvsControl_SE.xls

Tab-delimited text file can be opened in Excel. The file format definition is as follows:

column	names	description
1	GeneID	AS event in gene
2	geneSymbol	geneSymbol,'NA' for none
3	chr	chromosome ID
4	strand	strand specificity
5	exonStart_0base	start position of SE event (the position that 1 nt upstream of the 5' end of the skipped exon)
6	exonEnd	end position of SE event (the 3' end position of skipped exon)
7	upstreamES	the start position of upstream exon in SE event
8	upstreamEE	the end position of upstream exon in SE event
9	downstreamES	the start position of downstream exon in SE event
10	downstreamEE	the end position of downstream exon in SE event
11	IC_SAMPLE_1	inclusion junction counts for SAMPLE_1, with only the reads span splicing junctions taken into account. Biological replicates are seperated by comma.
12	SC_SAMPLE_1	exclusion junction counts for SAMPLE_1, with only the reads span splicing junctions taken into account.
13	IC_SAMPLE_2	inclusion junction counts for SAMPLE_2, which calculation method is the same as (12)
14	SC_SAMPLE_2	exclusion junction counts for SAMPLE_2, which calculation method is the same as (13)

15	IncFormLen	length of inclusion form, used for normalization
16	SkipFormLen	length of skipping form, used for normalization
17	PValue	the p-value
18	FDR	the adjusted p-value
19	IncLevel1	inclusion level for SAMPLE_1 replicates (comma separated) calculated from normalized counts
20	IncLevel2	inclusion level for SAMPLE_2 replicates (comma separated) calculated from normalized counts
21	IncLevelDifference	average(IncLevel1) - average(IncLevel2)

3.2 TreatvsControl_A5SS.xls / TreatvsControl_A3SS.xls

Tab-delimited text file can be opened in Excel. The file format definition is as follows:

column	names	description
1-4		1-54columns are with the same format as in TreatvsControl_SE.xls
5	longExonStart_0base	The start position of AS (the position that 1 nt upstream of the 5' end of the longer exon)
6	longExonEnd	The end position of AS (the 3' end position of the longer exon)
7	shortES	The position that 1 nt upstream of the 5' end of the shorter alternative-spliced exon after AS event
8	shortEE	The end position of the shorter alternative-spliced exon after AS event
9	flankingES	the start position that 1 nt upstream of the 5' end of the exon dirctly next to the splice site
10	flankingEE	the end position that 1 nt upstream of the 5' end of the exon directly to the splicing side
11-21		11-21 columns are with the same format as in TreatvsControl_SE.xls

3.4 TreatvsControl_MXE.xls

Tab-delimited text file can be opened in Excel. The file format definition is as follows:

column	names	description
1-4		1-4 columns are with the same format as in TreatvsControl_SE.xls
5	1stExonStart_0base	The start position of MSE (the position that 1 nt upstream of the 5' end of the first skipped exon (which locates in the upstream of the second exon))
6	1stExonEnd	The end position of MSE (the 3' end position of the first skipped exon)
7	2ndExonStart_0base	The start position of MSE (the position that 1 nt upstream of the 5' end of the second skipped exon)
8	2ndExonEnd	The end position of MSE（the end position of the second skipped exon)
11-21		11-21 columns are with the same format as in TreatvsControl_SE.xls

3.5 TreatvsControl_RI.xls

Tab-delimited text file can be opened in Excel. The file format definition is as follows:

column	names	description
1-4		1-4 columns are with the same format as in TreatvsControl_SE.xls
5	riExonStart_0base	The start position of RI (the position that 1 nt upstream of the 5' end of the retained exon) (which
6	riExonEnd	The end position of RI (the 3' end position of the retained exon)
7-21		7-21 columns are with the same format as in TreatvsControl_SE.xls

3.6 TreatvsControl_SE/MXE/A5SS/A3SS/RI.significant.xls

Tab-delimited text file can be opened in Excel. The columns are with the same format as in TreatvsControl_SE/MXE/A5SS/A3SS/RI.xls, respectively, except that events with FDR < 0.05 regarded as significant event are retained.
